# The temporal build-up of hummingbird/plant mutualisms in North America and temperate South America

**DOI:** 10.1186/s12862-015-0388-z

**Published:** 2015-06-10

**Authors:** Stefan Abrahamczyk, Susanne S. Renner

**Affiliations:** Department of Biology, Nees Institute for Plant Biodiversity, University of Bonn, Meckenheimer Allee 170, 53113 Bonn, Germany; Department of Biology, Institute for Systematic Botany and Mycology, University of Munich (LMU), Menzinger Str. 67, 80638 Munich, Germany

**Keywords:** Coevolution, Chronograms, Hummingbirds, Patagonia, *Sephanoides*, Staggered clade ages

## Abstract

**Background:**

The 361 species of hummingbirds that occur from Alaska to Patagonia pollinate ~7,000 plant species with flowers morphologically adapted to them. To better understand this asymmetric diversity build-up, this study analyzes the origin of hummingbird/plant mutualisms in North America and temperate South America, based on new compilations of the 184 hummingbird-adapted species in North America, the 56 in temperate South America, and complete species-level phylogenies for the relevant hummingbirds in both regions, namely five in temperate South America and eight in North America. Because both floras are relatively well sampled phylogenetically, crown or stem ages of many representative clades could be inferred. The hummingbird chronogram was calibrated once with fossils, once with substitutions rates, while plant chronograms were taken from the literature or in 13 cases newly generated.

**Results:**

The 184 North American hummingbird-adapted species belong to ca. 70 lineages for 19 of which (comprising 54 species) we inferred divergence times. The 56 temperate South American hummingbird-adapted species belong to ca. 35 lineages, for 17 of which (comprising 25 species) we inferred divergence times. The oldest hummingbirds and hummingbird-adapted plant lineages in the South American assemblage date to 16–17 my, those in the North American assemblage to 6–7 my. Few hummingbird-pollinated clades in either system have >4 species.

**Conclusions:**

The asymmetric diversity build-up between hummingbirds and the plants dependent on them appears to arise not from rapid speciation within hummingbird-pollinated clades, but instead from a gradual and continuing process in which independent plant species switch from insect to bird pollination. Diversification within hummingbird-pollinated clades in the temperate regions of the Americas appears mainly due to habitat specialization and allopatric speciation, not bird pollination per se. Interaction tanglegrams, even if incomplete, indicate a lack of tight coevolution as perhaps expected for temperate-region mutualisms involving nectar-feeding vertebrates.

**Electronic supplementary material:**

The online version of this article (doi:10.1186/s12862-015-0388-z) contains supplementary material, which is available to authorized users.

## Background

Plants adapted for pollination by hummingbirds possess a syndrome of correlated traits, namely abundant sucrose-rich nectar, scentless, often brightly colored flowers, no landing platform, and stigmas and stamens placed such that foraging hummingbirds effect cross-pollination [1–5]. At least 84 % of hummingbird nectar flowers are red [6]. The required correlated trait changes have originated many times, and from Alaska to Tierra del Fuego, some 7000 species in 404 genera from 68 families now depend on one or more of the 361 species of hummingbirds for their pollination [7]. In western North America alone, bird pollination is thought to have arisen over 100 times [8], although the basis for this estimate is unclear. Inferring how fast and how often plant lineages became specialized for hummingbird pollination by acquiring ‘pro bird’ and ‘anti bee’ traits [5], requires clock-dated phylogenies for hummingbirds and the plant lineages adapted to them. Two studies have taken this approach. The first focused on the Neotropical Acanthaceae genus *Ruellia*, which has 350 species, half of them adapted for pollination by hummingbirds [9]. Hummingbirds evolved in the late Oligocene [9, 11] long before New World *Ruellia*, which originated 8.3 to 13.5 mya. This mismatch led Tripp and McDade [9] to suggest that *Ruellia* diversification was facilitated by a pre-existing diversity of hummingbirds. The second study focused on a section of *Passiflora* with 62–64 species, ~90 % of them pollinated by hummingbirds, especially the Sword-billed hummingbird, *Ensifera ensifera* [10]. The *Passiflora* clade has a similar age as *E. ensifera*, namely ca. 11 my, and its diversification apparently resulted from rapid evolution in small isolated populations in the high Andes [10].

**Fig. 1:**
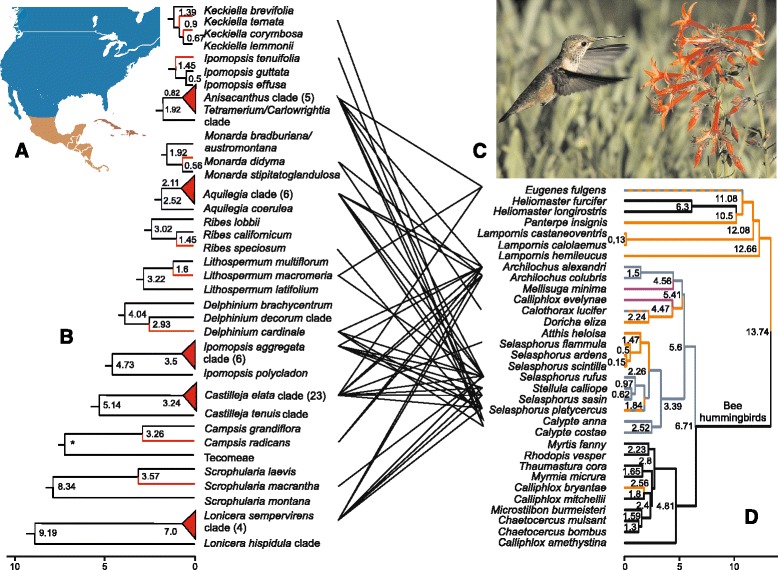
Tanglegrams for North American hummingbird species with plant species adapted for bird pollination (lines only connect plants and birds with empirical data on their interaction; see Table 1); **a**: Map of North America (*blue*), Central America (*orange*), and the Caribbean (lilac); **b**: Schematic depiction of 13 of the 19 dated North American hummingbird-pollinated clades (all 19 are in Table 1 but here we only include plants for which the name of their pollinating hummingbird species is known). Red lines indicate hummingbird-pollinated species, red triangles hummingbird-pollinated plant clades (species number in brackets). Stem ages and crown ages except for *Campsis* (marked by an asterisk) for which no stem age is provided in the original publication; **c**: *Selasphorus rufas* at *Ipomopsis aggregata* (Polemoniaceae), photo by M. Manske, Oregon Department of Transportation, www.wikipedia.org; **d**: Dated phylogeny and ancestral area reconstruction for Bee hummingbirds and Mountain Gems. North American species and lineages are indicated with blue lines, Central American species/lineages with orange, Caribbean species with lilac, and hummingbird lineages from other regions with black lines. Orange-blue dashed lines for *Calothorax lucifer*, *Eugenes fulgens,* and *Selasphorus platycercus* indicate that these species occur in Central and North America. Error margins on plant time estimates are shown in Table 1, those for birds in Tables S1. Time scales below figures are in million years before present

**Fig. 2:**
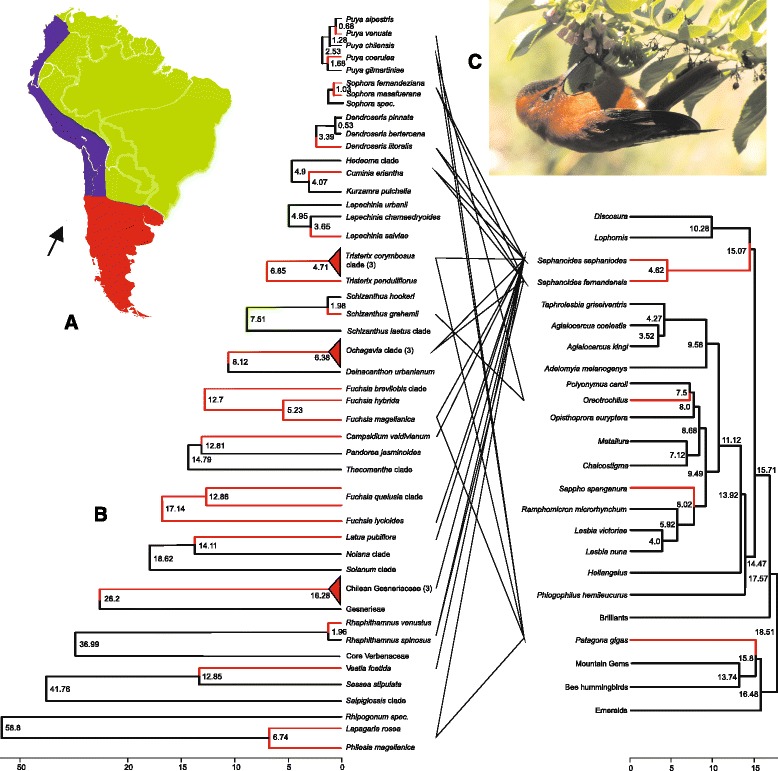
Tanglegrams for temperate South American hummingbird species with plant species adapted for bird pollination (lines only connect plants and birds with empirical data on their interaction; see Table 2); **a**: Map of temperate South America (*red*), tropical South America (*green*), and the Northern Andes (lilac); the arrow marks the Juan Fernandez Islands. B: Temporal build-up of temperate South American hummingbird-pollinated clades; red lines indicate hummingbird-pollinated species, red triangles hummingbird-pollinated plant clades (species number in brackets). Stem ages, and for clades also crown ages, are shown. C: *Sephanoides fernandensis* at *Cuminia eriantha* (Lamiaceae), photo by Héctor Gutiérrez Guzmán, www.wikipedia.org; D: Dated phylogeny for hummingbirds with the species occurring in temperate South America indicated by red lines. Error margins on plant age estimates are shown in Table 2, those for birds in Table S2. Time scales below figures are in million years before present

Here, we compare the diversity build-up in hummingbird/plant assemblages in North America (the region north of 24 °N; Fig. 1) and temperate South America (south of 27°S; Fig. 2), focusing on plants morphologically adapted for hummingbird pollination. We chose these two regions because of the tractable numbers of bird and plant species involved, available data on which bird species pollinate which plant species, and their ecological comparability, yet evolutionary independence. As the basis for our study, we compiled lists of both the bird-adapted plant species and the hummingbird species in both regions. North America has 18 species of hummingbirds ([12] our Additional file 5: Table S1) of which eight belong to the Bee hummingbirds and are almost endemic in North America. Temperate South America has six species of hummingbirds, one (*Rhodopis vesper*) barely extending its range from the tropics into the temperate climate ([13] our Table S2). There is no overlap in the native hummingbirds or the native plant species or genera between North America and temperate South America.

We expected the oldest North American bird/plant pollination mutualisms to be younger than the oldest South American ones because the North American birds appear to be younger. This is apparent from a molecular clock-dated hummingbird phylogeny that includes 284 species [11] and which indicates that the crown age of the Bee hummingbird clade to which most of the North American hummingbirds belong is just 5.3 Ma old. The temperate South American bird species, by contrast, have older divergence times, up to 14.4 my [11]. Nevertheless, old pollinator groups can pollinate young plant groups, and old plant clades can have young pollinators [9, 14–16]. Our comparative investigation at the species or (small) genus level differs from previous analyses, such as the above-mentioned study by Tripp and McDade [9] because we consider specific interacting plant and hummingbird species, using tanglegrams.

The main questions we wanted to answer were: (i) In each of the two biogeographic regions, are the oldest hummingbird species or clades and the oldest plant species or clades that depend on bird pollination of the same age? (ii) If so, is the entire North American bird/plant assemblage indeed younger than the temperate South American assemblage as expected from the younger ages of the North American birds or is there a temporal disconnect between bird and plant crown ages as in the case of *Ruellia*? And (iii) was the build-up of bird/plant mutualisms in the two regions gradual or instead temporarily clustered?

## Material and methods

### Plant taxon sampling and sequence alignment

We compiled all temperate North and South American plant species reported as pollinated by hummingbirds and/or showing morphological and physiological adaptations to hummingbird pollination, such as: (i) brightly colored, scentless flowers, with (ii) long, often quite stiff corolla tubes, (iii) exposed stigmas and stamens, (iv) large amounts of sucrose-rich nectar, and (v) semi-pendent exposed position, and (usually) no landing platform [2, 5]. Plant species occasionally visited by hummingbirds, but mainly pollinated by insects were not included.

For three clades (*Aquilegia, Lithospermum*, *Lonicera*), we used published divergence time estimates (*Results*), while for 13 others we downloaded and modified or newly compiled alignments from GenBank (www.ncbi.nlm.nih.gov/genbank/) or TreeBASE (www.treebase.org/). For North America, we used alignments that included bird-pollinated species of *Castilleja* (Orobanchaceae) from [17]; *Keckiella* (Plantaginaceae), modified from [18]; *Monarda* (Lamiaceae), newly built; *Ipomopsis* and *Collomia* (Polemoniaceae) from [19]; and *Ribes* (Grossulariaceae) from [20]. For temperate South America, we used alignments that included species of *Campsidium* (Bignoniaceae) modified from [21]; *Vestia* (Solanaceae) from [22]; *Dendroseris* (Asteraceae) from [23]; *Puya* (Bromeliaceae) from [24]; *Rhaphithamnus* (Verbenaceae) modified from [25]; *Schizanthus* (Solanaceae) modified from [26]; *Latua* (Solanaceae) modified from [27]; and *Tristerix* (Loranthaceae) from [28]. The taxonomic composition of each alignment, gene regions used, and alignment lengths are listed in Additional file 5: Table S3, which also shows the GenBank accession numbers of species added to some of the alignments.

### Plant clock models, their calibration and cross validation

Molecular clock dating of the 13 matrices relied on BEAST version 1.7.5 [29], with strict and relaxed clock models applied to each matrix. Relaxed clock models were preferred if the ucld.stdev value in Tracer version 1.6.0 [30] was ≥0.5 (ucld.stdev values for each matrix are reported in Table S3). All Markov chain Monte Carlo (MCMC) runs employed a Yule tree prior and the GTR + G substitution model with four rate categories (as in most of the original studies cited in the previous section). MCMC chains were run for 20 million generations, sampling every 10,000^th^ generation, unless stated otherwise in Table S3. Of the posterior trees, we dropped the first 20 % as burn-in and then checked convergence, using Tracer, making sure that all effective sample sizes (ESS) were >300. ESS values indicate the number of effectively independent draws from the posterior in the sample, and this statistic can help to identify autocorrelation and poor mixing. Tree Annotator (part of the BEAST package) was then used to create maximum clade credibility trees. Trees were visualized, edited and rooted in Fig Tree [31]. Error bars (95 % confidence intervals) are only shown for nodes having a posterior probability ≥ 98 %.

For calibration we used a range of published nuclear or plastid substitution rates or (in two cases) secondary calibrations from other studies with taxonomically overlapping nodes as listed for each matrix in Table S3. We validated each calibration by comparing the age of at least one node in each of our chronograms with the age of the same node in published fossil-calibrated chronograms, such as the angiosperm-wide study of Bell et al. [32] or other studies as listed in Table S3.

### Bird taxon sampling and sequence alignment

Our alignment comprised five species of swifts and 221 species of hummingbirds of which 151 came from the alignment of McGuire et al. [33], 58 were downloaded from GenBank (Table S4), 9 were provided by McGuire (now included in [11]), and three (*Trochilus polytmus, T. scitulus,* and *Cyanophaia bicolour*) came from [34]. The 18 hummingbird species that occur in North America (Table S1) are all in the matrix, but of the temperate South American species we lack *Oreotrochilus leucopleurus*, one of six species in this genus, three of them included in the matrix. The mitochondrial and nuclear regions used in the bird alignment are listed in Table S4, which also gives the GenBank accession numbers of the downloaded sequences added to the alignment using the Mesquite software [35]. The concatenated final matrix had 4022 aligned positions and 15.1 % empty cells. The matrix and a maximum likelihood tree have been submitted to TreeBASE (accession number S17392).

### Bird clock models, their calibration and cross validation

Molecular clock dating of the hummingbird matrix relied on BEAST with the same basic strategy as used for the plant dating. Modeltest (http://www.hiv.lanl.gov/content/sequence/findmodel/findmodel.html) gave the GTR + G substitution model, followed by the HKY + G model, as best fitting the mitochondrial data (226 x 1977 aligned nucleotides). The best-fitting model for the nuclear matrix of 2045 aligned nucleotides, excluding empty cells (missing sequences), was the K2P + G model. For the combined matrix (4022 nucleotides) we chose a substitution model of intermediate complexity, namely the HKY + G model. A relaxed clock model fit the combined data less well than a strict clock (ucld.stdev value = 0.16). We therefore used a strict clock model, and calibrated it with a hummingbird stem group fossil from the oil shale of Messel in Southern Germany that provides a minimum age for the divergence between hummingbirds and swifts [36]. Argon dating of igneous rocks underlying the Messel pit indicates a Lower Lutetian age of approximately 47.5 my [37], which has been used for fossils from this pit. We used a gamma distribution with an off-set at 47.5 my, a shape parameter of 2.1, and a median of 51.37, allowing 95 % of the ages to fall between 48.1 and 60.0 my, and 2.25 % to be older than 60.0 my (Additional file 1: Fig. S1a). This encompasses the stem age of 58.5 my obtained by Bleiweiss [38].

As an alternative to the fossil calibration, we used an uncorrelated lognormal (UCLN) relaxed clock (ucld.stdev values for the nuclear genes: 0.635, for the mitochondrial genes: 0.213) and calibrated it using a mean mitochondrial substitution rate of 0.0112 substitutions/site/year x 10^−6^, which has been calculated for hummingbirds using geographic and habitat-age calibrations [39]. This rate is in line with the general bird mitochondrial substitution rate of 2 % (sequence divergence rate/2 = substitution rate [40, 41]). For the nuclear sequences, BEAST calculated a rate of 0.025 substitutions/site/year x 10^−6^ (Additional file 2: Figs. S1b). This fits with nuclear rates in animals being considerably slower than mitochondrial rates [42].

### Pollinator state reconstructions

Species were coded as hummingbird pollinated based on the data cited in our own lists (*Results*) or as pollinated by bees, flies, butterflies or moths based on relevant studies e.g., [18, 43, 44]. In a few cases, pollination mode was inferred from flower color, flower size and orientation, corolla tube length, and the time when flowers are open (see the criteria in the section *Plant taxon sampling and sequence alignment*). We then used the plant chronograms to infer the origin of hummingbird pollination using ancestral state reconstruction under maximum likelihood optimization in BayesTraits 1.3 [45] or under parsimony optimization in Mesquite 2.75 [35]. Most nodes that we accepted as hummingbird pollinated had probabilities of >70 % for that state; *Aquilegia* and *Lonicera* had values between 65 and 70 %.

### Biogeographic analyses

This study focuses on hummingbirds and plants in North America north of 24°N (which includes northernmost Mexico) and in temperate South America south of the Atacama Desert (south of 27°S). These latitudes represent the border between the temperate and the subtropical habitats where many tropical species reach their northern- or southernmost distribution. After excluding four of the five swift outgroup species, each hummingbird species was assigned to one of the following biogeographic regions: North America, temperate South America, Central America, West Indies, tropical South American lowlands, and Northern Andes, based on Schuchmann [13] and Williamson [12]. Ancestral area reconstruction relied on Bayesian Binary MCMC analysis as implemented in RASP 2.1beta [46, 47]. As input trees, we used 2,001 trees from the fossil-calibrated BEAST run and deleted all outgroup species except for *Streptoprocne zonalis*. We used 50,000 iterations, sampling every 100^th^, with the Jukes-Cantor + G model of state transitions. *Streptoprocne zonalis* was assigned as outgroup using the “custom” option. All switches between areas and area combinations were allowed except that we permitted maximally two areas for the root node because a larger ancestral range seemed implausible. We only report inferred ancestral areas for North America and temperate South America and only those that had likelihoods ≥75 %.

## Results

### The ages of the interacting bird and plant species/clades in the two geographic regions

At least 184 North American (Table S5) and 56 temperate South American plant species (Table S6) are pollinated by hummingbirds as documented by field observations or in a few cases inferred from the floral traits associated with hummingbird pollination and listed in *Materials and Methods* (Tables 1, 2, and S5 and S6 provide references). The 184 species go back to at least 70 ancestors, the 56 to at least 35, numbers obtained by counting the genera in Tables S5 and 6 and adding the number of independent transitions to hummingbird pollination within *Penstemon* (Plantaginaceae; [18]) and *Lithospermum* (Boraginaceae, cf. chronogram with pollinator coding and inferred transitions in Additional file 3: Figure S2e). For *Ipomopsis* (Polemoniaceae; chronogram and inferred transitions in Additional file 3: Fig. S2c) we are unsure how often hummingbird pollination evolved because the analysis in BayesTraits yielded no unambiguous pollinator state for the crown node, while a parsimony analysis yielded hummingbird pollination.

**Table 1:** The 19 North American hummingbird-adapted plant species/clades, their family assignment, stem and/or crown ages, and pollinators. Numbers in parentheses after plant genera refer to the number of species in the alignment, followed by the total species in the respective clade. Full chronograms are in the online supporting materials Figs. S2a-h

Split of species or clade from nearest relative in alignment	Family	Stem age (my)	Crown age (my)	Age reference; figure number	Pollinators	Pollinator reference
*Anisacanthus* clade (5/5; *A. andersonii, A. quadrifidus, A. linearis, A. puberulus, A. thurberi*)	Acanthaceae	1.92 (0.21-4.27)	0.82	Cortes, 2013; Fig. 1	*Amazilia berryllina, Amazilia violiceps, Archilochus alexandrei, Calypte costae, Calothorax lucifer, Chlorostilbon canivetii, Cynanthus latirostris, Eugenes fulgens, Hylocharis leucotis, Selasphorus platycercus, Selasphorus rufus*	Daniel, 1982, Dennis & Tekulsky, 1991, Williamson, 2001, van Devender et al., 2004; Holmquist et al., 2005
*Aquilegia* clade (5/9; *A. canadensis, A. elegantula, A. flavescens, A. formosa, A. skinneri*)	Ranunculaceae	2.52 (NA)	2.11	Bastida et al., 2010, Figs. 1 and S2a	*Archilochus alexandri, Archilochus colubris, Selasphorus platycercus, Selasphorus rufus, Selasporus sasin*	Dennis & Tekulsky, 1991, Williamson, 2001, google pictures search: Bretzke Lane webside 06.03.2014
*Arbutus peninsularis*	Ericaceae	0.81 (NA)	-	Hardy & Cook, 2012	*Hylocharis xantusii*	Williamson, 2001
*Campsis radicans*	Bignoniaceae	3.26 (NA)	-	Xiang et al. 2000; Fig. 1	*Archilochus alexandrei, Archilochus colubris*	Williamson, 2001
*Castilleja elata* clade (16/54; *C. affinis*, *C. applegatei, C. chromosa, C. elata, C. elmeri, C. hispida, C. integrifolia, C. integra, C. linariifolia, C. miniata, C. minor, C. peckiana, C. peirsonii, C. praeterita, C. pruinosa, C. tenuiflora*)	Orobanchaceae	5.14 (3.36-6.92)	3.24 (2.14-4.34)	Figs. 1 and 2b	*Archilochus alexandri, Archilochus colubris, Amazilia violiceps, Calothorax lucifer, Eugenes fulgens, Hylocharis leucotis, Selasphorus flammula, Selasporus platycercus, Selaphorus rufus, Selasphorus sasin, Stellula calliope*	James, 1972, Williamson, 2001, van Devender et al., 2004, Lara-Rodriguez et al., 2012
*Collomia rawsoniana*	Polemoniaceae	2.12 (0.37-3.87)	-	Fig. S2c	Unknown	
*Delphinium cardinale*	Ranunculaceae	2.93 (NA)	-	Jabbour & Renner 2012; Fig. 1	*Archilochus alexandrei, Calypte costae Selasphorus platycercus, Selasporus rufus*	Schuchmann, 1999, Williamson, 2001
*Ipomopsis aggregata* clade (8/8; *I. aggregata ssp. aggregata, I. aggregata ssp. attenuata, I. aggregata ssp. bridgesii, I. aggregata ssp. collina, I. aggregata ssp. formosissima, I. arizonica, I. rubra, I. sancti-spiritus*)	Polemoniaceae	4.73 (NA)	3.5	Figs. 1 and S2c	*Archilochus alexandri, Selasporus platycercus, Selasporus rufus,* *Selasphorus sasin, Stellula calliope*	Carpenter, 1978, Schuchamnn, 1999, Williamson, 2001
*Ipomopsis tenuifolia*	Polemoniaceae	1.49 (0.21-3.10)	-	Figs. 1 and S2c	*Calpyte costae*	Wood & Nakazato, 2009
*Keckiella cordifolia*	Plantaginaceae	1.19 (0.29-2.09)	-	Fig. S2d	Unknown	
*Keckiella ternata* clade (2/3; *K. corymbosa, K. ternata*)	Plantaginaceae	1.39 (0.56-2.22)	0.9 (0.32-1.48)	Figs. 1 and S2d	*Calypte anna*	Williamson, 2001
*Lithospermum johnstonii*	Boraginaceae	2.52 (NA)	-	Cohen, 2012; Fig. S2e	Unknown	
*Lithospermum leonotis*	Boraginaceae	0.96 (NA)	-	Cohen, 2012; Fig. S2e	Unknown	
*Lithospermum macromeria*	Boraginaceae	1.58 (NA)	-	Cohen, 2012; Figs. 1 and S2e	*Eugenes fulgens, Selasphorus rufus*	Boyd, 2004
*Lithospermum notatum* clade (2/2; *L. flavum, L. notatum*)	Boraginaceae	8.36 (NA)	5.11	Cohen, 2012; Fig. S2e	Unknown	
*Lonicera sempervirens* clade (4/4; *L. arizonica, L. ciliosa, L. dioica, L. sempervirens*)	Caprifoliaceae	9.19 (4.8-13.05	7.0 (3.67-10.60)	Smith & Donoghue, 2010; Figs. 1 and 2f	*Archilochus alexandrei, Archilochus colubris, Selasphorus platycercus, Selasphorus rufus, Selasphorus sasin, Stellula calliope*	Dennis & Tekulsky, 1991, Williamson, 2001
*Monarda didyma*	Lamiaceae	0.56 (NA)	-	Figs. 1 and 2g	*Archilochus colubris, Selasphorus rufus*	Schuchmann, 1999, Williamson, 2001
*Ribes speciosum*	Grossulariaceae	1.45 (0.10-3.07)	-	Figs. 1 and 2h	*Calypte anna*	Stiles, 1973
*Scrophularia macrantha*	Scrophulariaceae	3.57 (0.65-7.61)	-	Scheunert & Heubl 2011; Fig. 1	*Selasphorus rufus*	Schuchmann, 1999

**Table 2:** The 17 temperate South American hummingbird-adapted plant species/clades, their family assignment, stem and/or crown ages, and pollinators. Numbers in parentheses after clade names refer to the number of species in the alignment. Full chronograms are in the online supporting materials Figs. S3a-h. The very long stem lineage of Philesiaceae (crown age 6.7 my, stem age 58.8 my), a Southern Chilean family of two species, is explained by its closest living relative species in Australia (with 52 my old macrofossils in Tasmania [64])

Split of species or clade from nearest relative in alignment	Family	Stem age (my)	Crown age (my)	Age reference; figure number	Pollinators	Pollinator reference
*Campsidium valdivianum*	Bignoniaceae	12.8 (NA)	-	Figs. 2, S3a	*Sephanoides sephanoides*	Aizen & Vazquez, 2006
Chilean Gesneriaceae (3; *Asteranthera ovata, Mitraria coccinea*, *Sarmienta repens*)	Gesneriaceae	26.2 (NA)	16.28 (NA)	Woo et al., 2011; Fig. 2	*Sephanoides sephanoides*	Aizen & Vazquez, 2006
*Cuminia eriantha*	Lamiaceae	4.07 (NA)	-	Drew & Systma, 2012; Fig. 2	*Sephanoides fernandensis, Sephanoides sephanoides*	Bernadello et al., 2001
*Dendroseris litoralis*	Asteraceae	3.4 (NA)	-	Figs. 2, S3b	*Sephanoides fernandensis*, *Sephanoides sephanoides*	Schuchmann, 1999
*Fuchsia lycioides*	Onagraceae	17.14 (NA)	-	Berry et al., 2004; Fig. 2	*Rhodopsis vesper*, *Sephanoides sephanoides*	Atsatt & Rudel (1982), Reid et al. (2002) mentions that *S. sephaiodes* and *F. lycioides* occur in the same locality
*Fuchsia magellanica*	Onagraceae	5.23 (NA)	-	Berry et al., 2004; Fig. 2	*Patagona gigas, Sephanoides sephanoides*	Smith-Ramirez, 1993, Belmonte Schwarzbaum, 1999
*Latua pubiflora*	Solanaceae	14.11 (NA)	-	Figs. 2, S3c	*Sephanoides sephanoides*	Based on plant distribution
*Lepechinia salviae*	Lamiaceae	3.65 (NA)	-	Drew & Sytsma, 2013; Fig. 2	-	-
*Ochagavia clade* (3/4; *Fascicularia bicolor, O. carnea, O. elegans*)	Bromeliaceae	8.12 (NA)	6.38 (NA)	Givnish et al., 2013; Fig. 2	*Sephanoides sephanoides Sephanoides fernandensis*	Roy et al., 1998, Medan & Montaldo, 2005
*Philesia magellanica*, *Lapagaria rosea*	Philesiaceae	58.8 (NA)	6.74 (NA)	Chacón & Renner, 2014; Fig. 2	*Patagona gigas*, *Sephanoides sephanoides*	Belmonte Schwarzbaum, 1999, Aizen & Vazquez, 2006
*Puya coerulea*	Bromeliaceae	1.68 (NA)	-	Figs. 2, S3d	*Patagona gigas*; *Oreotrochilus leucopleurus*	Jabaily & Sytsma, 2010; Hornung et al., 2013
*Puya venusta*	Bromeliaceae	0.68 (NA)	-	Figs. 2, S3d	*Patagona gigas*	Jabaily & Sytsma, 2010
*Rhaphithamnus venustus*	Verbenaceae	1.96 (NA)	-	Figs. 2, S3e	*Sephanoides sephanoides*, *Sephanoides fernandensis*	Smith-Ramirez, 1993, Schuchmann, 1999
*Schizanthus grahamii*	Solanaceae	1.98 (NA)	-	Figs. 2, S3f	*Oreotrochilus leucopleurus*	Perez et al., 2006
*Sophora fernandeziana * *S. masafuerana*	Fabaceae	1.03 (NA)	-	Ruiz et al., 2004; Fig. 2	*Sephanoides sephanoides Sephanoides fernandensis*	Bernadello et al., 2004
*Tristerix* (3; *T. aphyllus, T. corymbosus, T. verticillatus*)	Loranthaceae	6.85 (NA)	4.71 (NA)	Figs. 2, S3g	*Patagona gigas*, *Sephanoides sephanoides*	Smith-Ramirez, 1993; Amico et al., 2007
*Vestia foetida*	Solanaceae	12.85 (NA)	-	Figs. 2, S3h	*Sephanoides sephanoides*	Based on plant distribution

We were able to infer divergence times for 19 of the c. 70 independent North American hummingbird-adapted species/clades, and together they include 58 of the 184 hummingbird-adapted species (Table 1). For temperate South America, we were able to infer divergence times for 17 of the c. 35 independent origins of bird pollination, and together they include 25 of the 56 hummingbird-adapted species (Table 2). Figures 1 and 2 illustrate the temporal build-up for 13 of the 19 North American species/clades and for the 17 temperate South American species/clades, along with the divergence times of the birds that pollinate them. For six of the North American plants we had no information on the specific hummingbird species pollinating them (only that they are pollinated by hummingbirds is known: Table 1), and they could therefore not be included in the tanglegram.

The oldest North American hummingbird-adapted plants in our sample are the *Lonicera* clade (Caprifoliaceae; 4 of the 5 bird-pollinated species listed in Table S5 are included in the alignment, stem age 9.2 my, crown age 7.0 my; [48]) and the *Lithospermum notatum* species group (Boraginaceae; 2 species; stem age 8.3 my; crown age 5.1 my, Table 1). The oldest temperate South American hummingbird-adapted group in our sample is a Chilean Gesneriaceae clade of three species (*Asteranthera ovata, Mitraria coccinea*, *Sarmienta repens*, stem age 26.2 my, crown age 16.3 my, [49]). The youngest North American hummingbird-pollinated species in our sample is *Monarda didyma* (Lamiaceae; divergence from sister species at 0.6 my; Table 1), and the youngest temperate South American hummingbird adapted clade is *Puya venusta* (Bromeliaceae; divergence from sister species at 0.7 my; Table 2).

Our hummingbird chronogram (Additional file 1: Figs. S1a from the fossil-calibrated strict clock and Additional file 2: Fig. S1b from the rate-calibrated relaxed clock, with 95 % Highest Posterior Density [HPD] intervals) is similar to a chronogram from largely overlapping DNA data [11]. The ages inferred with the two calibrations differ little even for the hummingbird crown age (the deepest node), which is 24 (20.9-28.1) my using the fossil calibration or 25 (23–27) my using the rate calibration. Outside evidence supporting these dating efforts comes from several sources (*Discussion*).

In the fossil-calibrated chronogram, the North American Bee hummingbird radiation has a stem age of 6.8 my and a crown group age of 5.6 my (blue circle in Additional file 1: Fig. S1a). The Emeralds and Mountain Gems whose ranges extend from Central America into northern Mexico, Arizona and Texas (Additional file 5: Table S1) have similar ages as the North American Bee hummingbirds. However, the Mountain Gem, *Eugenes fulgens*, ranging from Costa Rica to southern Arizona, is older, dating to about 11.1 my (Figs. 1, Additional file 1: S1a, Additional file 5: Table S1). The temperate South American species, *Sephanoides sephaniodes* and *S. fernandensis* form a clade that has a stem age of 15.1 my and a crown age of 4.6 my (Figs. 2, Additional file 1: S1a, Additional file 5: Table S2). The other three species in temperate South America, *Patagona gigas, Sappho sparganura* and *Oreotrochilus leucopleurus*, are not closely related to each other. They are 15.8, 8.0, and between 7.5 and 2.5 my old (since we lack *O. leucopleurus* we assume that its age lies somewhere between the stem [7.5. my] and crown [2.5 my] age of the genus *Oreotrochilus*, of which we included three of its six species. *Rhodopis vesper,* barely extending into temperate South America (it occurs along coastal regions of Peru and Chile), is 2.2 my old (Figs. 2, Additional file 1: S1a, Additional file 5: TableS2).

The 7 my crown age and 9.2 my stem age of the oldest North American food-plant clade, *Lonicera*, more or less matches the age of *Eugenes fulgens* (11.1 my) and the stem age of the North American Bee hummingbird clade, 6.8 my. Similarly, the 16.3 my crown age of the oldest temperate South American food-plant clade more or less matches the stem ages of the genera *Sephanoides*, 15.1 my, and *Patagona*, 15.8 my (Figs. 2 and Additional file 1: S1a).

### The origin of the diversity asymmetry between bird species and bird-pollinated plant species

In North America, eight of the ~70 transitions to hummingbirds as pollinators (mostly in different genera; Additional file 5: Table S5) comprise five or more species (*Aquilegia, Castilleja, Ipomopsis, Lonicera*, *Mimulus*, *Penstemon*, *Salvia*, *Silene*). We here dated 19 of these 70 transitions with together 54 species (Table 1), with our sampling including the largest bird-pollinated genus, *Castilleja*, as well as some of single-species transitions to bird pollination. In temperate South America, none of the ~35 transitions to hummingbird pollination has resulted in a clade with >4 species; the three genera with at least four hummingbird-pollinated species are *Greigia, Lobelia,* and *Tristerix* (Additional file 5: Table S5b). We dated 17 of the 35 transitions to hummingbird pollination with together 25 species (Table 2). As shown in Figs. 1 and 2 (also Tables 1 and 2) the diversity build-up appears to have been gradual. Species numbers in 33 sister taxon pairs, with one member pollinated by hummingbirds, the other not (Additional file 5: Table S7), do not suggest a consistent positive effect of hummingbird pollination on diversification.

## Discussion

The main questions we wanted to answer concerned the timeframe of the bird/plant pollination mutualisms in North America and temperate South America, namely do the oldest plant species/clades and bird species/clades match in age? And was the build-up of bird/plant mutualisms in the two regions gradual or did it instead involve temporarily clustered radiations. Our results reveal that in each of the two regions, the oldest interacting clades are indeed of matching age, at least within the error of molecular clock dating and given our incomplete species sampling, but the North American bird/plant assemblage is roughly half as old as the temperate South American one, yet has more than 3x as many bird-pollinated plant species. The diversity build-up in both regions was gradual, rather than occurring in clustered radiations (below). The oldest temperate South American species are *Patagona gigas* and the genus *Sephanoides*, both perhaps 15 my old and thus much older than the Bee hummingbird clade in North America (stem age 6.8 my, crown group age 5.6 my). Other species that pollinate North American plants, such as a few Emeralds and Mountain Gems have low abundances in North America, and several of them have only extended their ranges northwards during the last 100 years [12, 50]. The younger age of the North American assemblage may be the footprint of more pronounced Pleistocene extinctions in that region.

Our study provides the fourth independent molecular-clock dating of hummingbird divergence times, and we used both fossil calibration and rate calibration. The crown group age we inferred with either calibration (24 or 25 my) is slightly older than the 18 my inferred by Bleiweiss [38] from DNA melting temperatures and a 28 species matrix, the 21 my inferred by Jetz et al. [51] from a phylogenetic tree that included 6,663 bird species, 233 of them hummingbirds, or the 22.4 my (20.3-24.7) from a tree that included 284 hummingbird taxa (some with multiple accessions) calibrated with substitution rates of Hawaiian honeycreepers [11]. Tripp and McDade [9], similar to us, calibrated the McGuire et al. [33] matrix with a fossil-based constraint at the swift/hummingbird split, but assigned the stem-group hummingbird fossil used for calibration to the crown node of hummingbirds. In our fossil-calibrated chronogram, the divergence between *Sephanoides fernandensis*, the only hummingbird endemic to the Juan Fernandez Islands (marked by an arrow in Fig. 2a), and its southern Andean sister species *Sephanoides sephaniodes* [52] is dated to 4.6 my, which agrees well with the age of 5.8 my of oldest island of the Juan Fernandez Archipelago [53], the archipelago in which *Sephanoides fernandensis* is endemic.

The list of North American hummingbird-adapted species compiled for this study (Table S5) includes 50–60 more species than previous compilations, namely Grant’s [54] list of 129 hummingbird-adapted species in Western North America and Williamson’s [12] list of 111 species for all of North America. We estimate some 70 transitions to hummingbird pollination, including ten in *Penstemon* alone [18], while a previous estimate was 100 independent transitions [8]. The latter number implies 30 more gains and losses of hummingbird pollination, perhaps mostly in poorly sampled groups, such as *Castilleja* or *Penstemon*. From our species lists (Tables S5 and 6), it can be seen that the North American bird-pollinated flora is dominated by temperate herbaceous lineages, such as *Aquilegia, Castilleja, Penstemon*, and *Silene*, while the South America bird-pollinated flora is dominated by species from semi-woody tropical Andean clades (*Bomarea*, *Fuchsia*, *Iochroma*, *Puya, Passiflora* [10, 24, 55]). In both regions, however, the diversity build-up on the plant side appears to have been gradual, with individual species adapting to hummingbirds in < <0.5 Ma and many co-occurring species ‘serviced’ by the same bird species ([56, 57] cf. tanglegrams Figs. 1 and 2).

Our study provides an absolute time frame for these two asymmetric build-ups of animal/plant assemblages (asymmetric because each assemblage has many more plant than bird species). In each region, some hummingbird species co-occur and feed on the same plant species, which is the situation described by Janzen as diffuse coevolution, defined as an array of interacting populations or species generating “a selective pressure as a group” ([58] p. 611). One-to-one interactions, however, also exist in both regions, for example, between *Castilleja coccinea* (Orobanchaceae) and *Archilochus colubris*, the only hummingbird species occurring in eastern North America [12, 59] and between the Chilean Gesneriaceae *Asteranthera ovata, Mitraria coccinea*, *Sarmienta repens*) and *Sephanoides sephaniodes* in central and southern Patagonia [60–62].

For North America, we inferred 36 instances of hummingbird pollination evolving within clades with bee pollination (see chronograms in Additional 3: Figs. S2 and Additional file 4: S3), but only in *Castilleja* did this switch lead to subsequent diversification of a hummingbird-pollinated clade. Usually, related hummingbird-pollinated species, such as *Silene virginica, S. regia*, and *S. rotundifolia* (Caryophyllaceae*)*, occur in different habitats but share the same pollinator (*Archilochus colubris* [63]). In temperate South America, switching from insects to birds happened at least 13 times. These results show that while hummingbirds have contributed to plant diversification, once a species is hummingbird-pollinated, further speciation is rare, perhaps because of the extensive gene flow mediated by these strong-flying pollinations. That hummingbird pollination per se is not a diversifying factor is also implied by our tabulation of 33 sister clades with and without hummingbird pollination (Additional file 5: Table S7). Grant and Grant [56] hypothesized that the reason for the limited diversification in hummingbird-adapted plants in North America might be the young age of these mutualisms. However, this is unlikely to be the sole explanation since the much older temperate South American hummingbird-dependent plants are similarly species-poor. Instead, hummingbird pollination in temperate regions may slow down population fragmentation and geographically small-scale speciation because these vertebrate pollinators maintain across-population gene flow.

## Conclusions

This study provides absolute time frames for the build-up of hummingbird/plant mutualisms in North America and temperate South America, and these time frames turn out to differ greatly. In both regions, plant groups successively entered the new adaptive zone ‘hummingbird pollination,’ but this mode of pollination then did not lead to rapid further speciation (*Castilleja* is the only really species-rich bird-pollinated clade in the temperate regions of the Americas). Temperate-region mutualisms involving nectar-feeding and migrating vertebrates are unlikely to involve one-to-one coevolution because no temperate zone hummingbird species can afford to completely rely on a single plant species for their nectar. This is also implied by our tanglegrams, incomplete as they are.

### Availability of supporting data

All the supporting data are included as additional files under: http://www.biomedcentral.com/bmcevolbiol/authors/instructions/researcharticle#formatting-supporting-data.

## Additional files

Additional file 1: Figure S1a. Chronogram for 221 species of hummingbirds, rooted on 5 species of swifts, based on 4022 nucleotides of nuclear and mitochondrial DNA (*Materials and Methods*) analyzed under a strict clock model calibrated with a 47.5 my-old hummingbird-like fossil (red star). Numbers above branches are node ages (my). The North American species are marked in blue, the southern South America species in red, and the blue circle marks the crown group of the North American clade. The stem age of *Oreotrochilus* is marked in red. The photo (by Steve Garvie, www.wikipedia.org) shows *Lophornis ornatus* at *Stachytarpheta* spec. (Verbenaceae) flowers. The map shows the biogeographic regions used in the ancestral area reconstructions.

Additional file 2: Figure S1b. Chronogram from the same matrix as used for Fig. S1a analyzed under a UCLN relaxed clock model calibrated with a mitochondrial substitution rate (*Materials and Methods*). Numbers above branches are node ages (my) and bars at nodes with ≥98 % posterior probability indicate the 95 % confidence intervals on the estimated times. The coloring of bird species is as in Fig. S1a.

Additional file 3: Figs. S2a-2 h. Plant chronograms for North American clades.

Additional file 4: Figs. S3a-h. Plant chronograms for temperate South American clades.

Additional file 5: Supplementary material. Table S1 North American hummingbird species, with geographic ranges and divergence times from Fig. S1a. Node ages are followed by 95 % confidence intervals in brackets. Focal species in bold. Table S2. Temperate South American hummingbird species, with geographic ranges and divergence times from Fig. S1a. Node ages are followed by 95 % confidence intervals in brackets. *Oreotrochilus leucopleurus* has not been sequenced, and for this species we used the stem age of *Oreotrochilus* as the oldest possible age of the species, which could be much younger. Table S3 .Plant matrices newly clock-dated and/or used for ancestral state reconstructions for this study, 8 from North America and 8 from temperate South American; the GenBank accession numbers of a few sequences added to certain alignments (as specified in the online supporting material) are listed at the end of this table. Table S4. Hummingbird sequences from GenBank added to the alignment of McGuire et al. (2007). AK1 = intron 5 in the nuclear *adenylate kinase* (AK1) gene (ca. 660 base pairs [bp]); NADH subunit 4 and 2 = mitochondrial *NADH dehydrogenase* subunits 4 and 2 (ND4 and ND2, ca. 900 and ca. 1050 bp); Bfib = intron 7 in the *beta fibrinogen* (Bfib) gene (ca. 1100 bp). Table S5. Hummingbird-adapted plant species from North America. *Ipomopsis aggregata* subspecies are treated as separate species. Clades or species for which divergence times have been inferred (as cited in Table 1 and shown in the online chronograms) are marked in red. References for this table and :Table S6 are listed below S6. Table S6. Hummingbird-adapted plant species from temperate South America. Clades or species for which divergence times have been inferred (as cited in Table 2 and shown in the online chronograms) are marked in red. Table S7 .Sister taxa in which one member of a pair is pollinated by hummingbirds, the other is not, together with their species numbers.
