# Supplementary figures and images for: The temporal build-up of hummingbird/plant mutualisms in North America and temperate South America
Source: BMC Evol Biol. 2015 Jun 10;15:104. doi: 10.1186/s12862-015-0388-z (PMC4460853; doi:10.1186/s12862-015-0388-z)

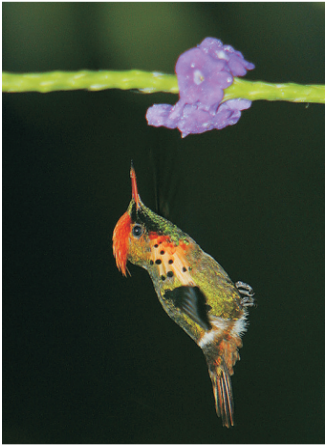

Fig. S1a

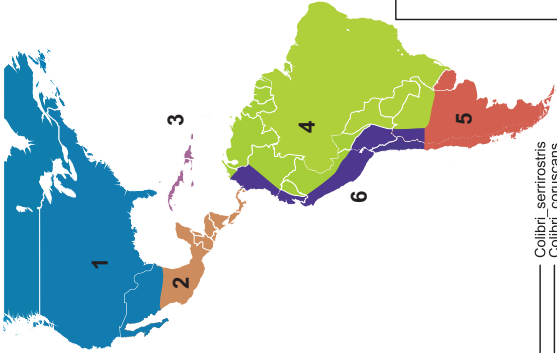

B  
A

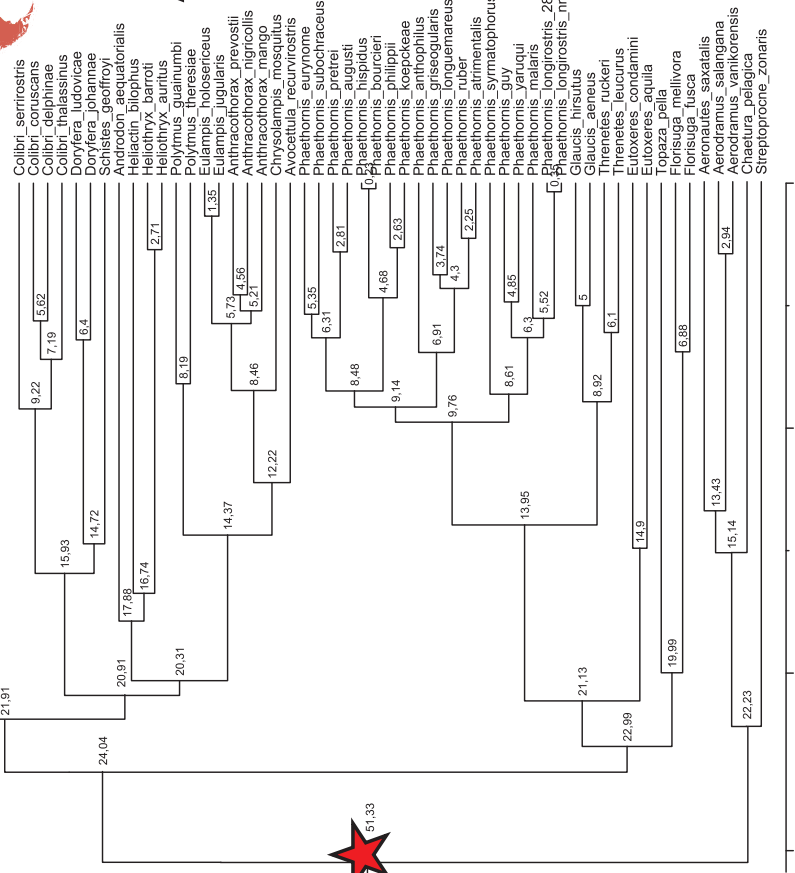

B

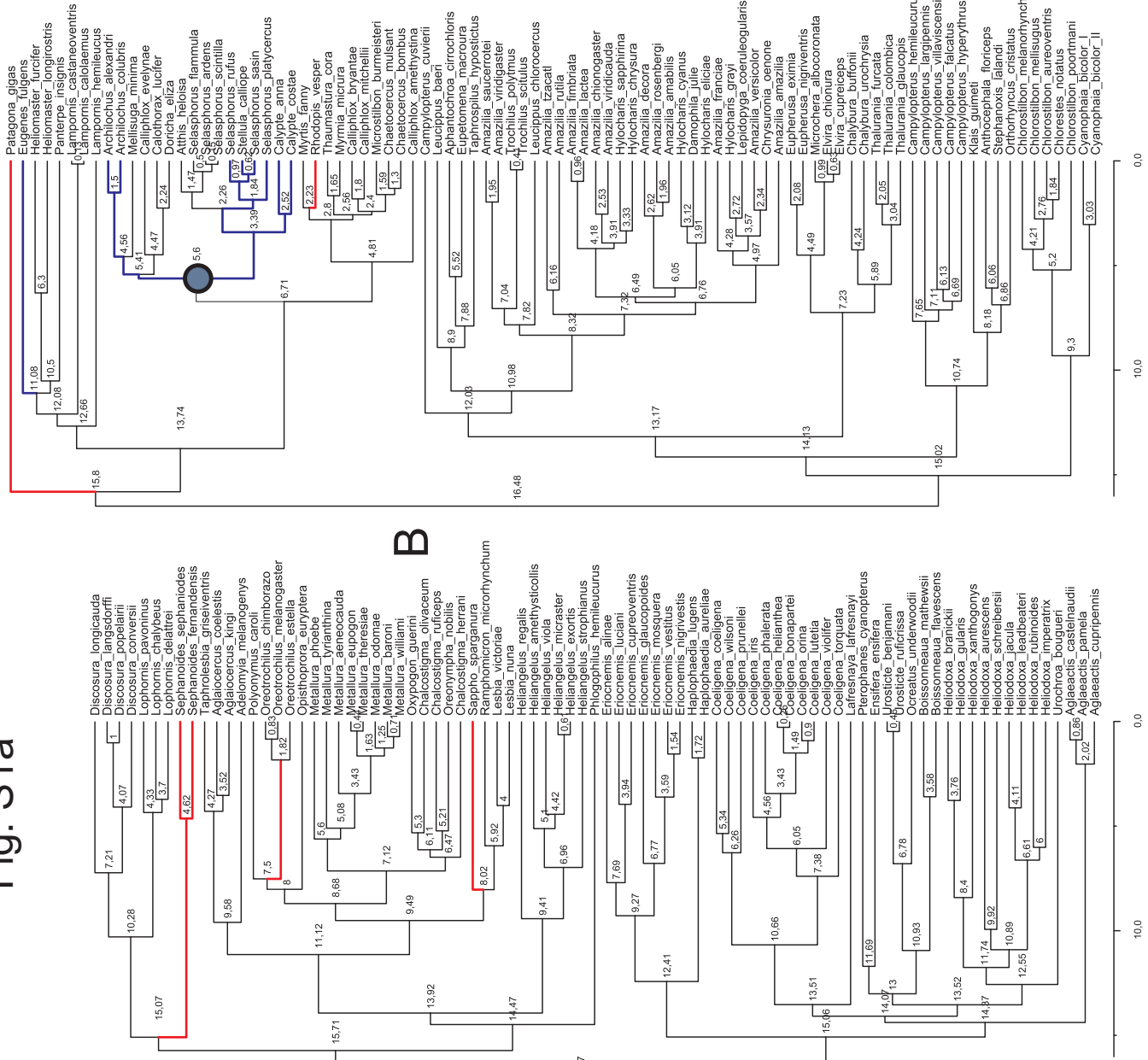

Supplement: Supplementary file 1 — Chronogram for 221 species of hummingbirds, rooted on 5 species of swifts, based on 4022 nucleotides of nuclear and mitochondrial DNA (Materials and Methods) analyzed under a strict clock model calibrated with a 47.5 my-old hummingbird-like fossil (red star). Numbers above branches are node ages (my). The North American species are marked in blue, the southern South America species in red, and the blue circle marks the crown group of the North American clade. The stem age of Oreotrochilus is marked in red. The photo (by Steve Garvie, www.wikipedia.org) shows Lophornis ornatus at Stachytarpheta spec. (Verbenaceae) flowers. The map shows the biogeographic regions used in the ancestral area reconstructions. [file 12862_2015_388_MOESM1_ESM.pdf]

**Fig. S1b**

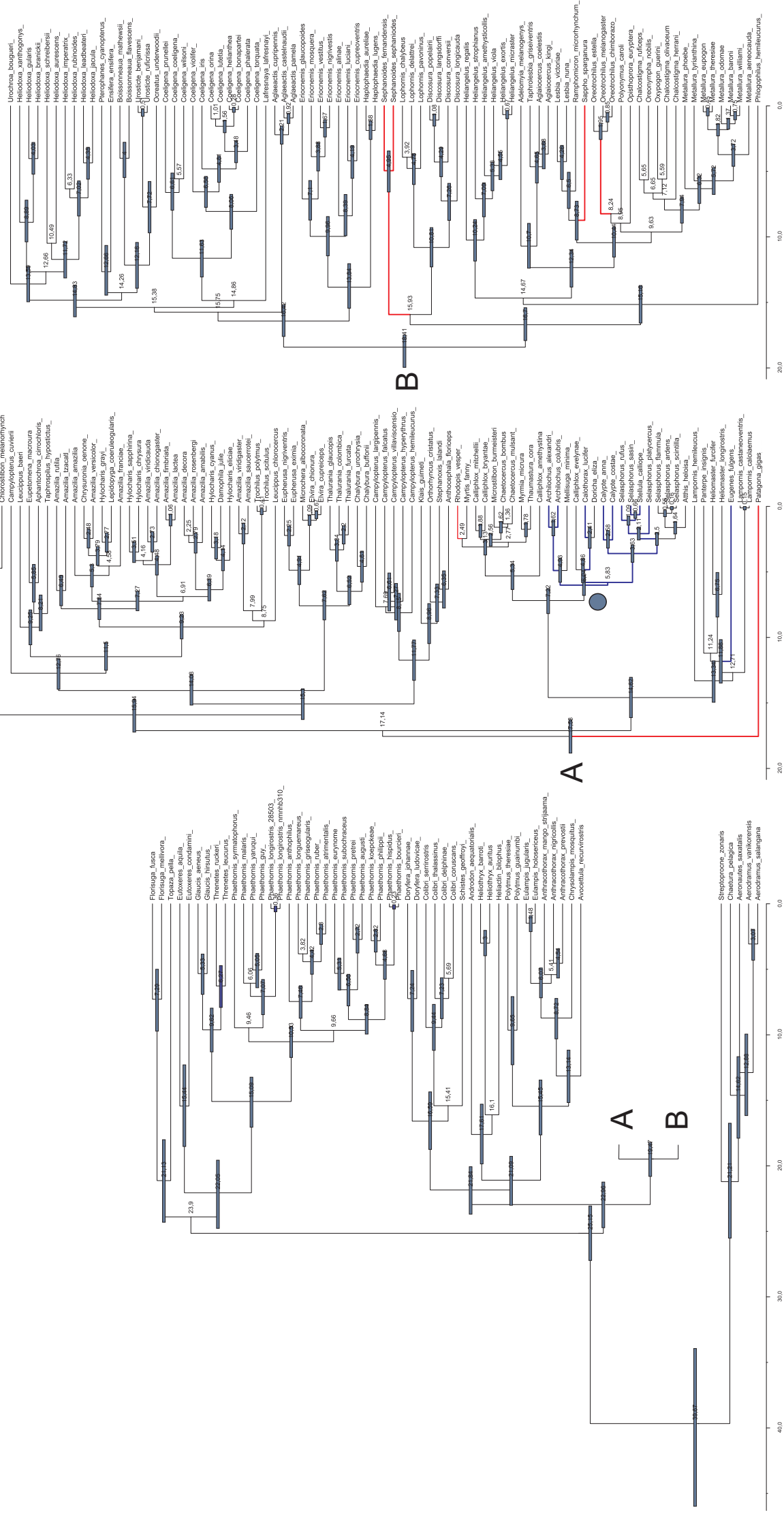

Supplement: Supplementary file 2 — Chronogram from the same matrix as used for Fig. S1a analyzed under a UCLN relaxed clock model calibrated with a mitochondrial substitution rate (Materials and Methods). Numbers above branches are node ages (my) and bars at nodes with ≥98 % posterior probability indicate the 95 % confidence intervals on the estimated times. The coloring of bird species is as in Fig. S1a. [file 12862_2015_388_MOESM2_ESM.pdf]
