# Supplementary material for: The temporal build-up of hummingbird/plant mutualisms in North America and temperate South America
Source: BMC Evol Biol. 2015 Jun 10;15:104. doi: 10.1186/s12862-015-0388-z (PMC4460853; doi:10.1186/s12862-015-0388-z)

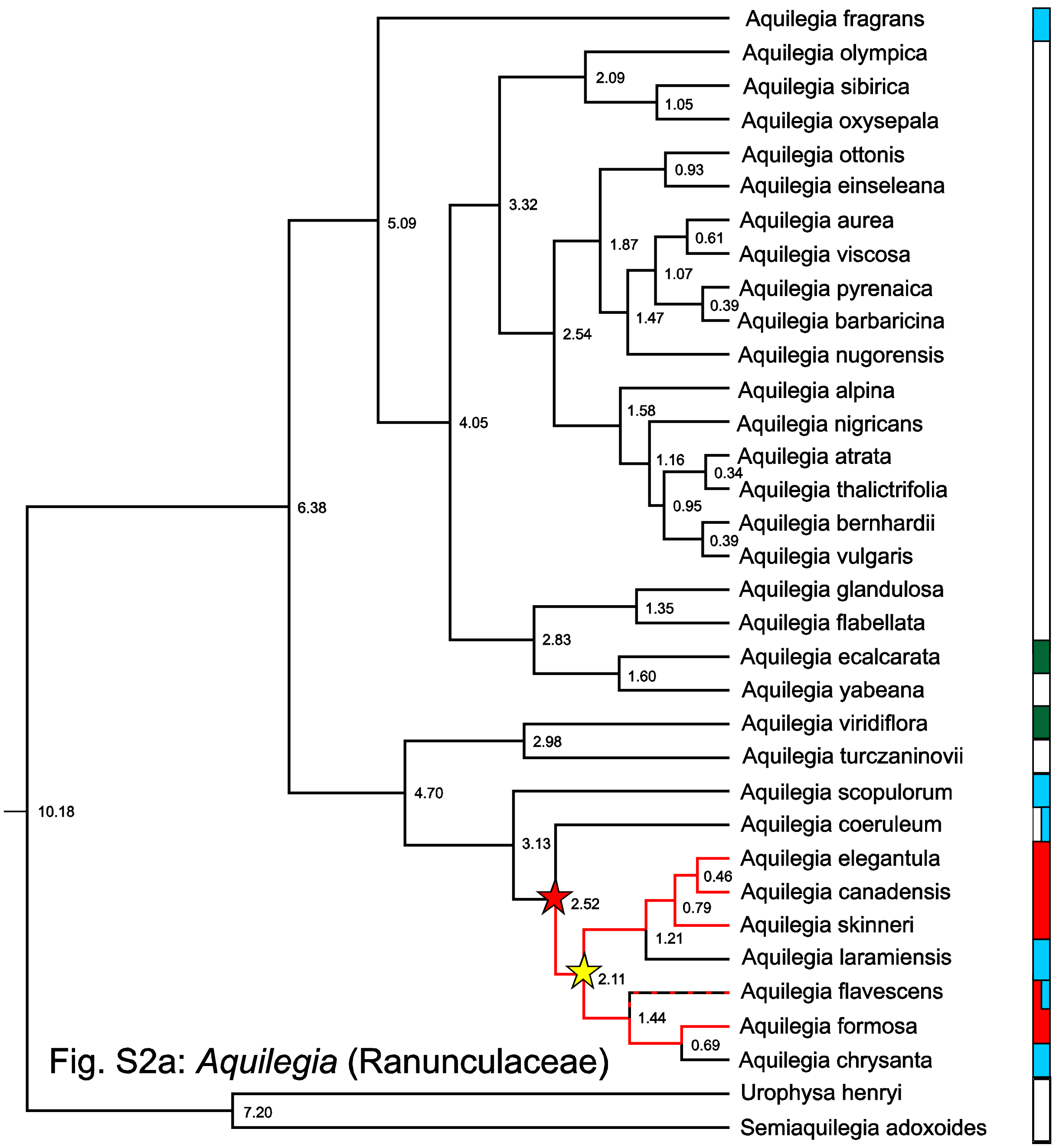

Fig. S2a: *Aquilegia* (Ranunculaceae)

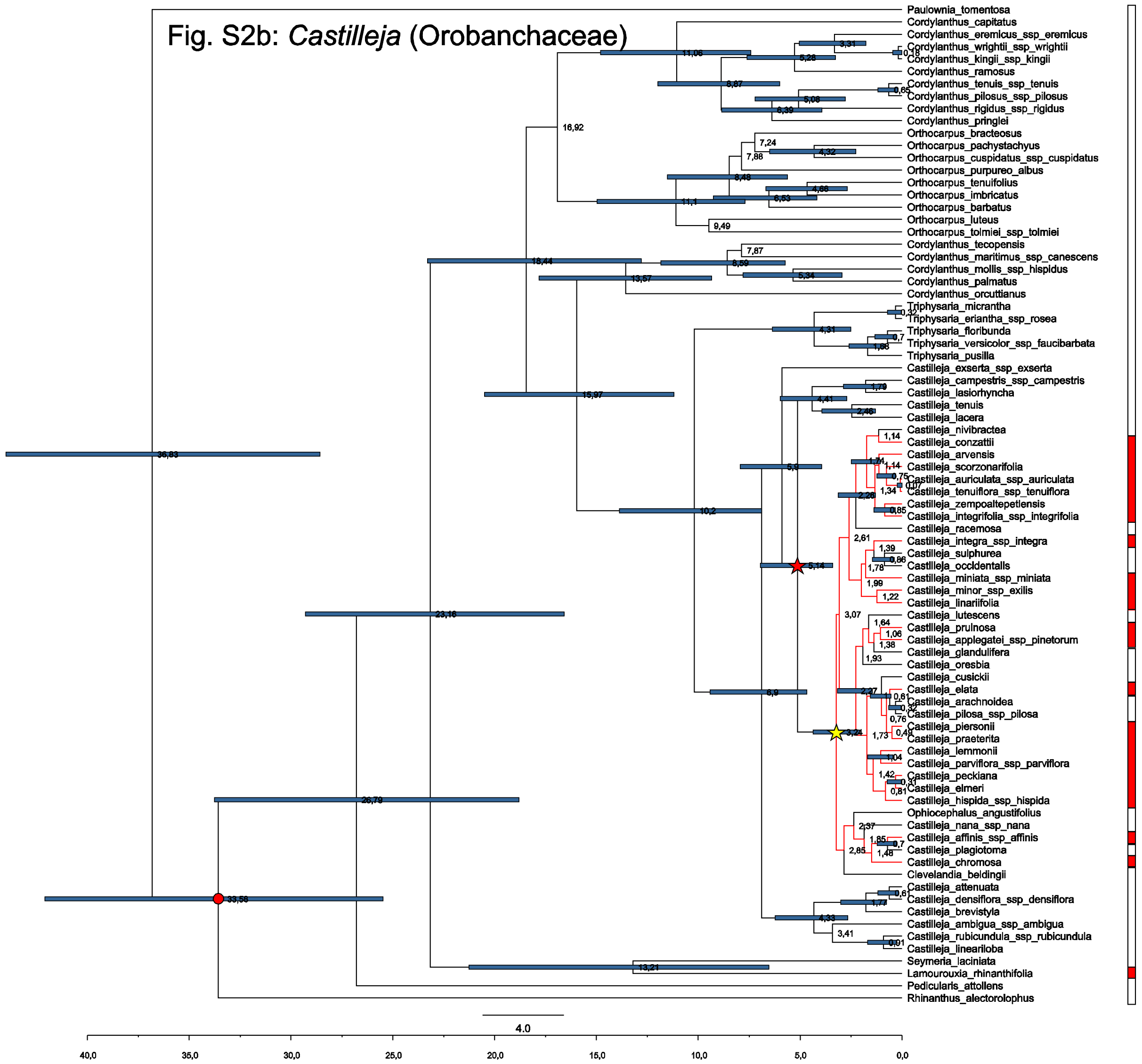

Fig. S2b: Castilleja (Orobanchaceae)
Paulownia_tomentosa
Cordylanthus_capitatus
Cordylanthus_eremicus_ssp_eremicus
Cordylanthus_wrightii_ssp_wrightii
Cordylanthus_kingii_ssp_kingii
Cordylanthus_ramosus
Cordylanthus_tenuis_ssp_tenuis
Cordylanthus_pilosus_ssp_pilosus
Cordylanthus_rigidus_ssp_rigidus
Cordylanthus_pringlei
Orthocarpus_bracteosus
Orthocarpus_pachystachyus
Orthocarpus_cuspidatus_ssp_cuspidatus
Orthocarpus_purpureo_albus
Orthocarpus_tenuifolius
Orthocarpus_imbricatus
Orthocarpus_barbatus
Orthocarpus_luteus
Orthocarpus_tolmiei_ssp_tolmiei
Cordylanthus_tecopensis
Cordylanthus_maritimus_ssp_canescens
Cordylanthus_mollis_ssp_hispidus
Cordylanthus_palmatus
Cordylanthus_orcuttianus
Triphysaria_micrantha
Triphysaria_eriantha_ssp_rosea
Triphysaria_floribunda
Triphysaria_versicolor_ssp_faucibarbata
Triphysaria_pusilla
Castilleja_exserta_ssp_exserta
Castilleja_campestris_ssp_campestris
Castilleja_lasiorhyncha
Castilleja_tenuis
Castilleja_lacera
Castilleja_nivibractea
Castilleja_conzattii
Castilleja_arvensis
Castilleja_scorzonerifolia
Castilleja_auriculata_ssp_auriculata
Castilleja_tenuiflora_ssp_tenuiflora
Castilleja_zempoaltepetlensis
Castilleja_integrifolia_ssp_integrifolia
Castilleja_racemosa
Castilleja_integra_ssp_integra
Castilleja_sulphurea
Castilleja_occidentalis
Castilleja_miniata_ssp_miniata
Castilleja_minor_ssp_exilis
Castilleja_linariifolia
Castilleja_lutescens
Castilleja_pruinosa
Castilleja_applegatei_ssp_pinetorum
Castilleja_glandulifera
Castilleja_oresbia
Castilleja_cusickii
Castilleja_elata
Castilleja_arachnoidea
Castilleja_pilosa_ssp_pilosa
Castilleja_piersonii
Castilleja_praeterita
Castilleja_lemmonii
Castilleja_parviflora_ssp_parviflora
Castilleja_peckiana
Castilleja_elmeri
Castilleja_hispida_ssp_hispida
Ophiocephalus_angustifolius
Castilleja_nana_ssp_nana
Castilleja_affinis_ssp_affinis
Castilleja_plagiotoma
Castilleja_chromosa
Clevelandia_beldingii
Castilleja_attenuata
Castilleja_densiflora_ssp_densiflora
Castilleja_brevistyla
Castilleja_ambigua_ssp_ambigua
Castilleja_rubicundula_ssp_rubicundula
Castilleja_lineariloba
Seymeria_laciniata
Lamourouxia_rhinanthifolia
Pedicularis_attollens
Rhinanthus_alectorolophus
4.0
40,0
35,0
30,0
25,0
20,0
15,0
10,0
5,0
0,0

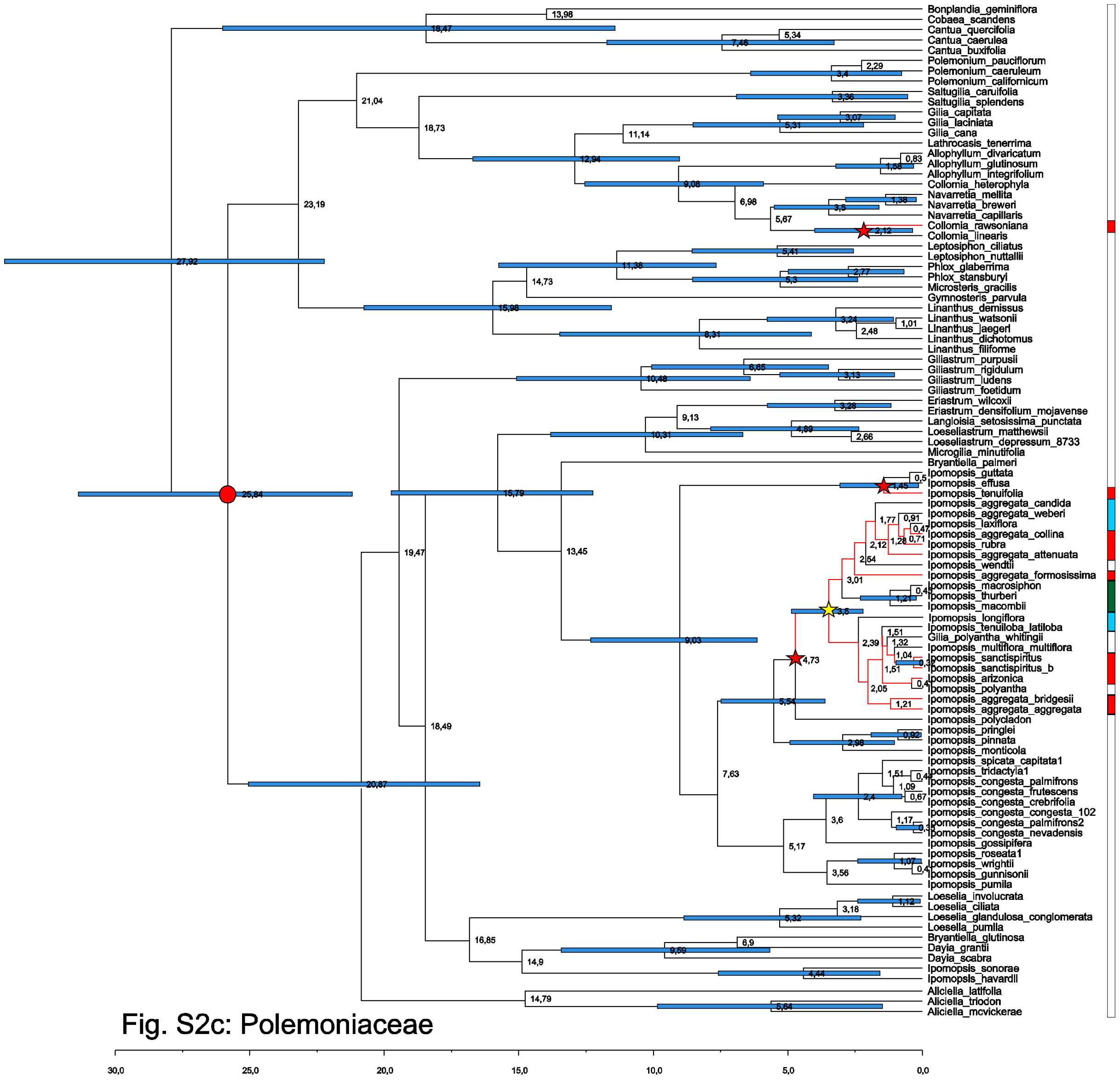

Fig. S2c: Polemoniaceae

Fig. S2d: *Keckiella* (Plantaginaceae)

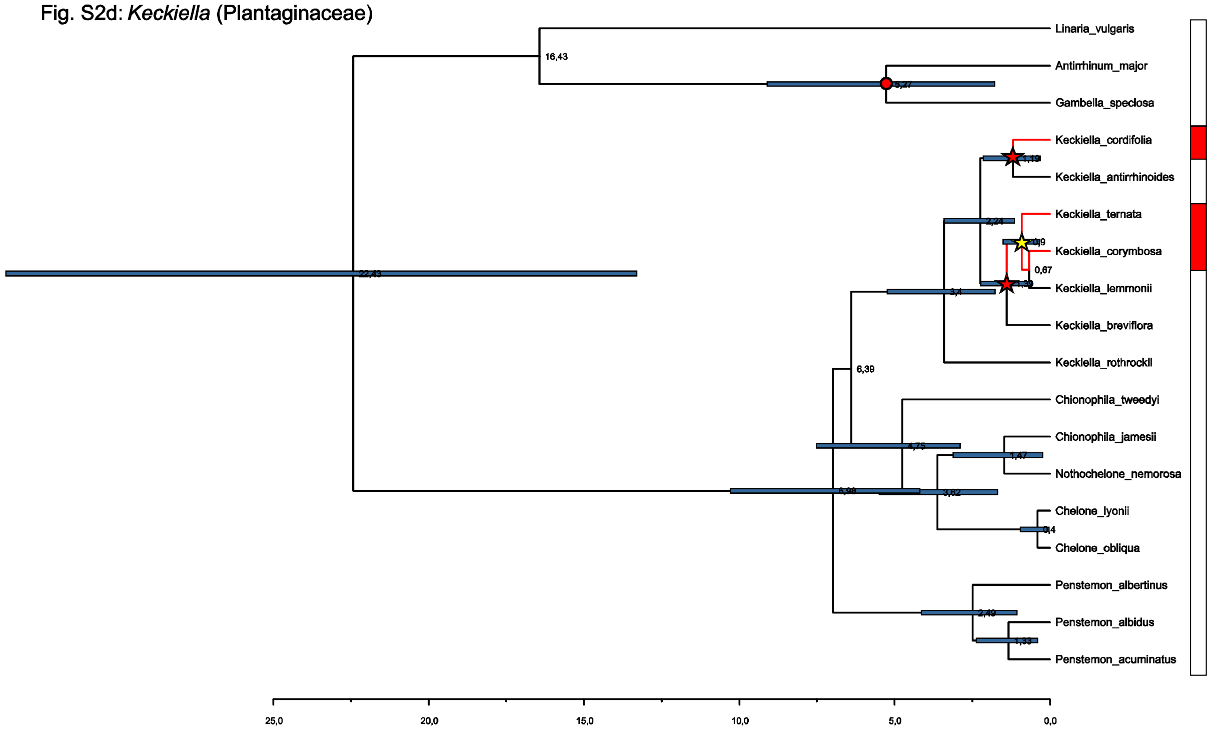

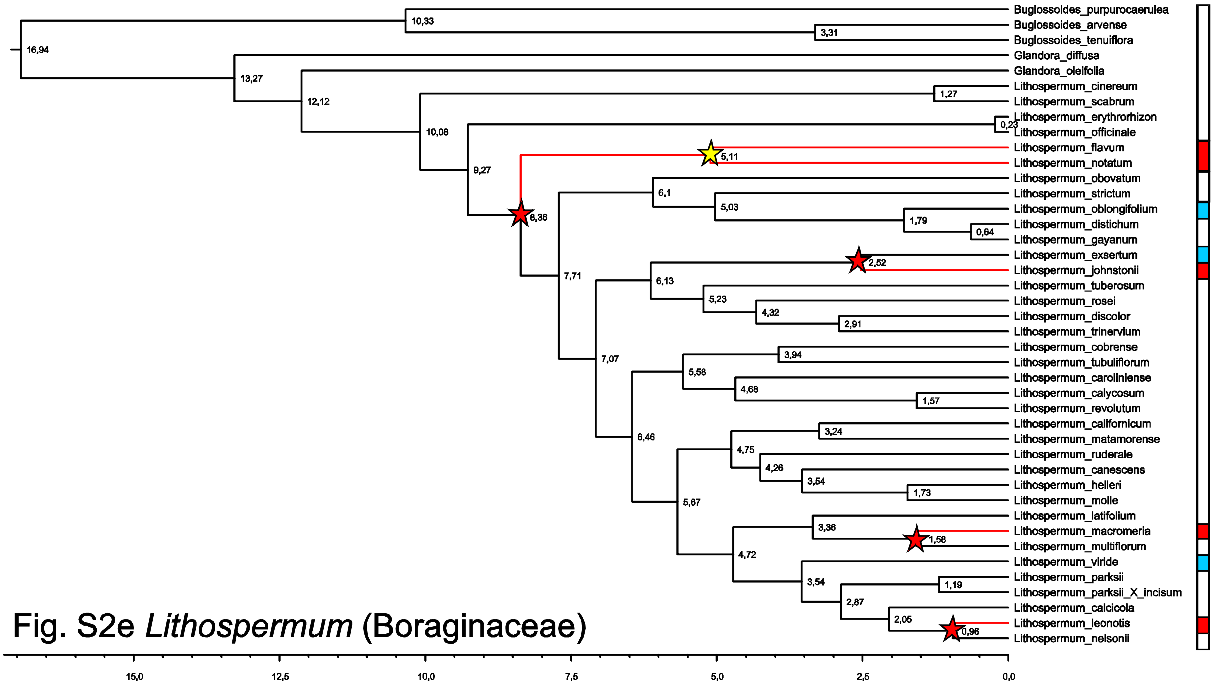

Fig. S2e *Lithospermum* (Boraginaceae)

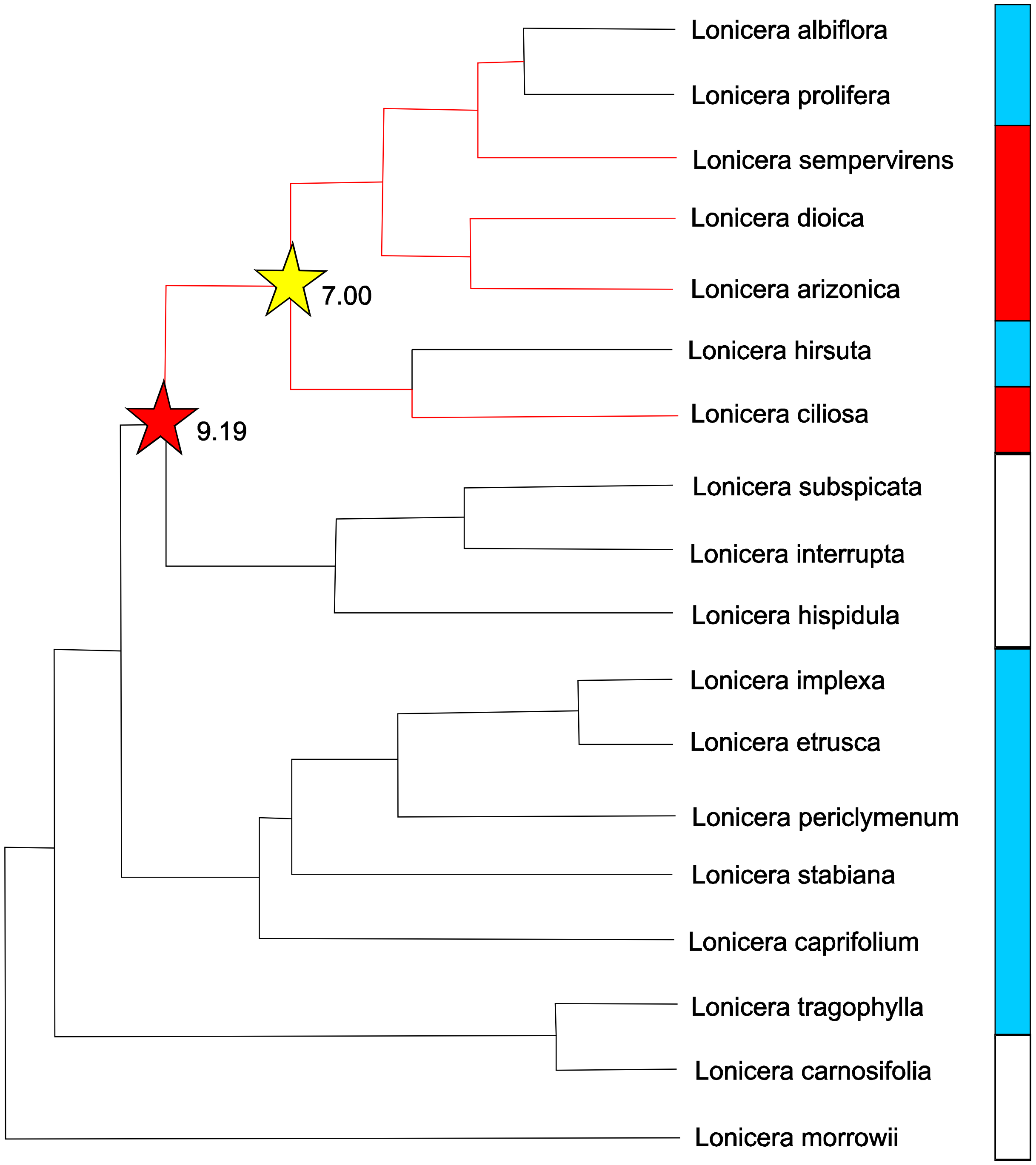

Fig. S2f *Lonicera* (Caprifoliaceae)

Fig. S2g *Monarda* (Lamiaceae)

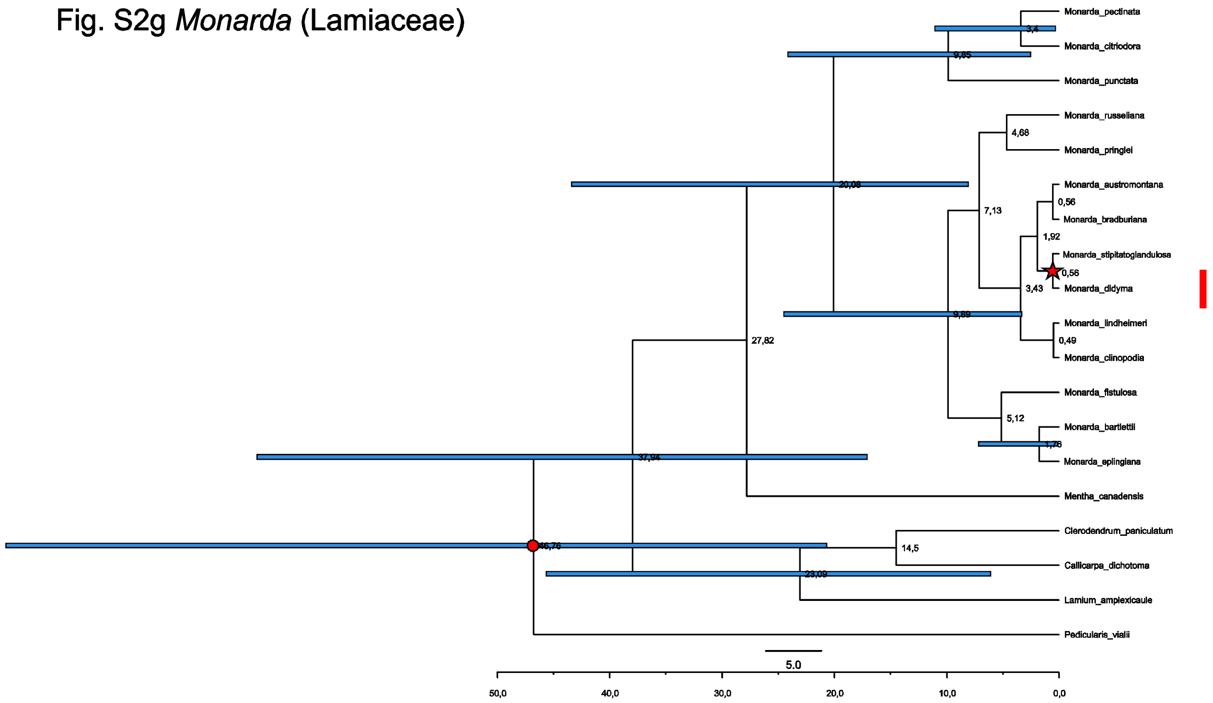

Fig. S2h: *Ribes* (Grossulariaceae)

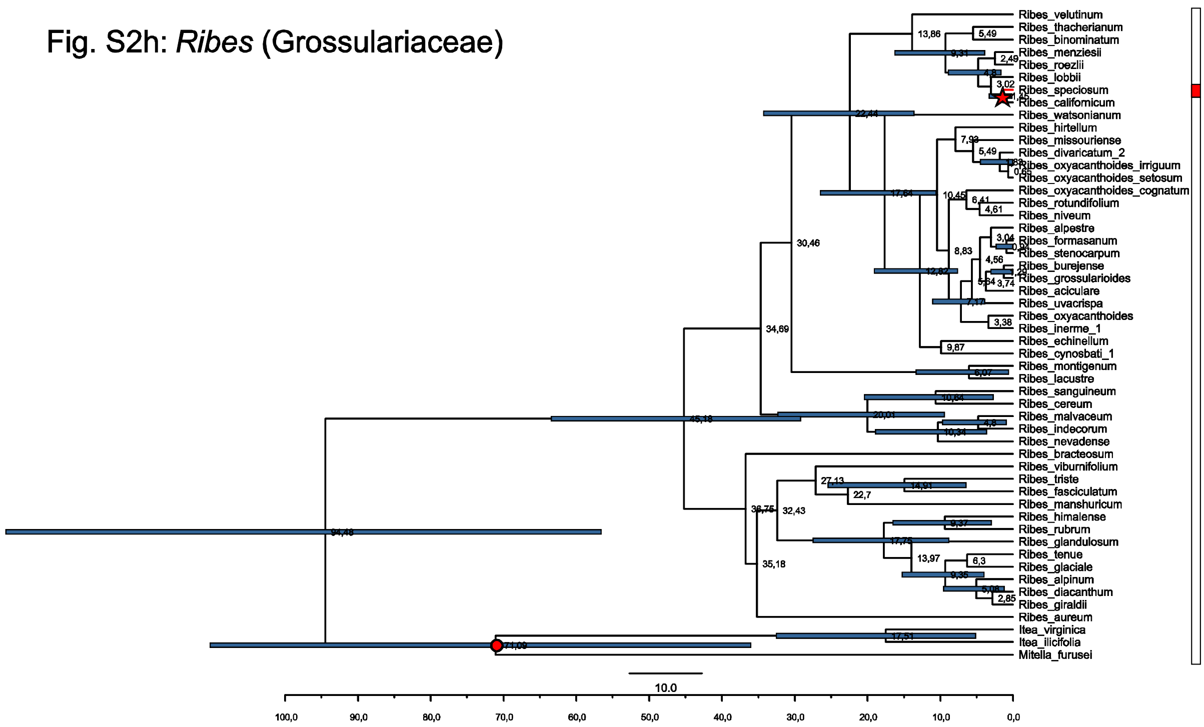

Supplement: Supplementary file 3 — Plant chronograms for North American clades. [file 12862_2015_388_MOESM3_ESM.pdf]
