# Supplementary material for: The temporal build-up of hummingbird/plant mutualisms in North America and temperate South America
Source: BMC Evol Biol. 2015 Jun 10;15:104. doi: 10.1186/s12862-015-0388-z (PMC4460853; doi:10.1186/s12862-015-0388-z)

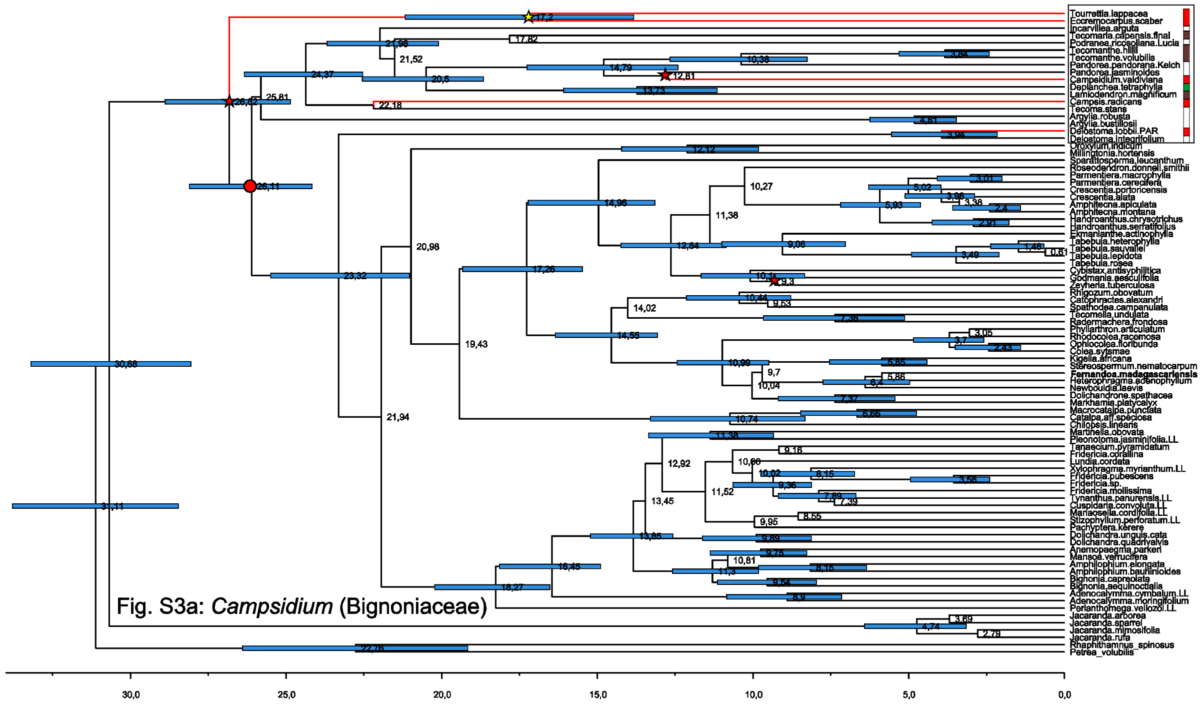

Fig. S3a: *Campsidium* (Bignoniaceae)

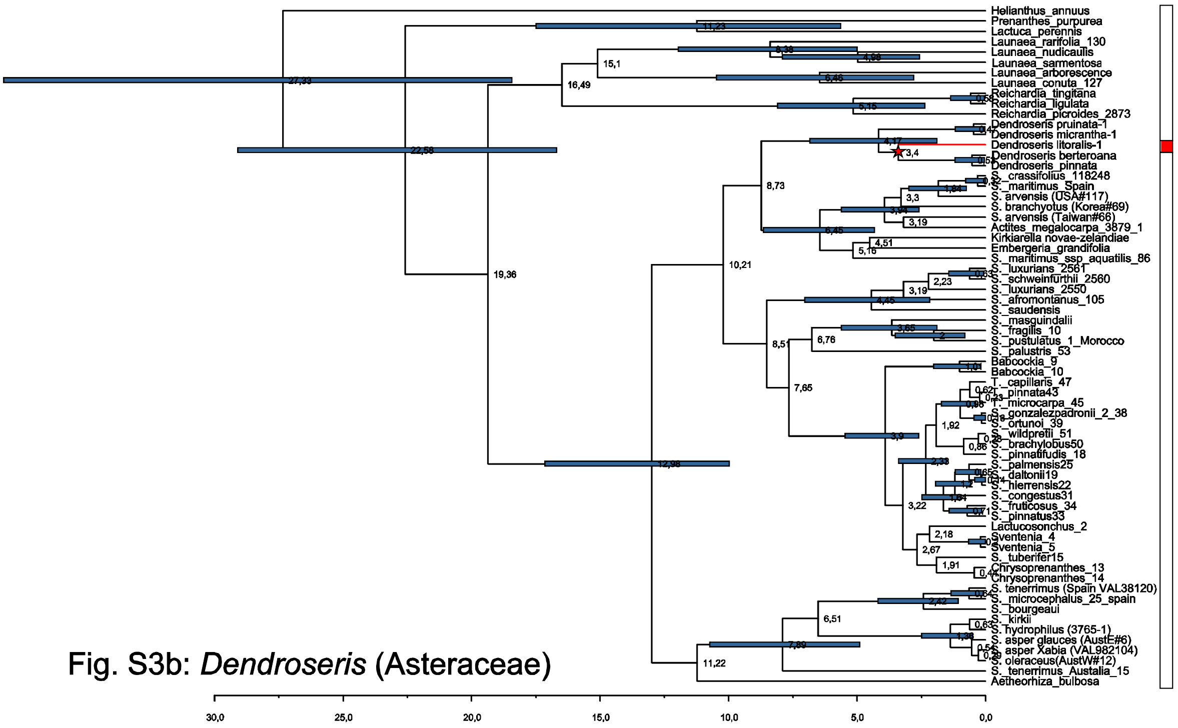

Fig. S3b: *Dendroseris* (Asteraceae)

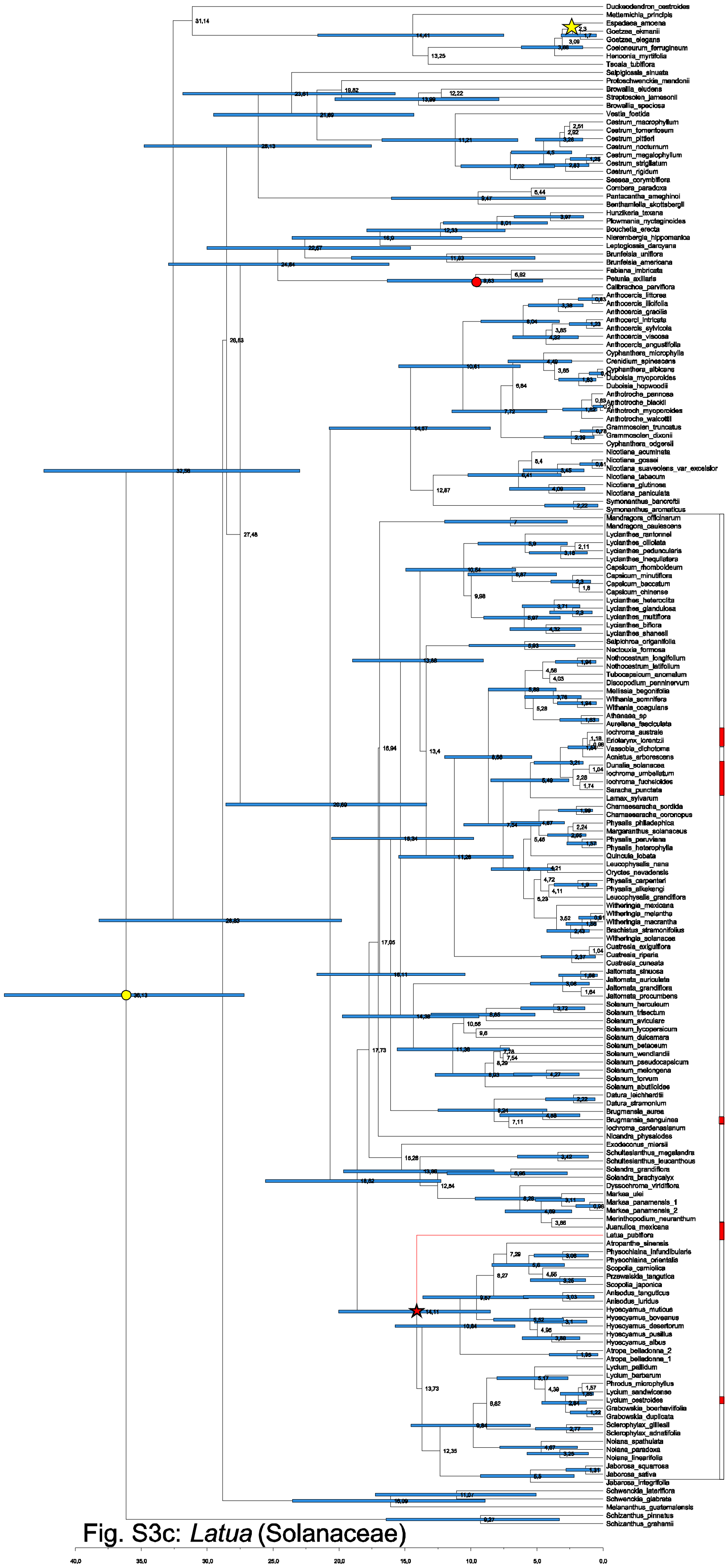

Fig. S3c: *Latua* (Solanaceae)

Fig. S3d: *Puya* (Bromeliaceae)

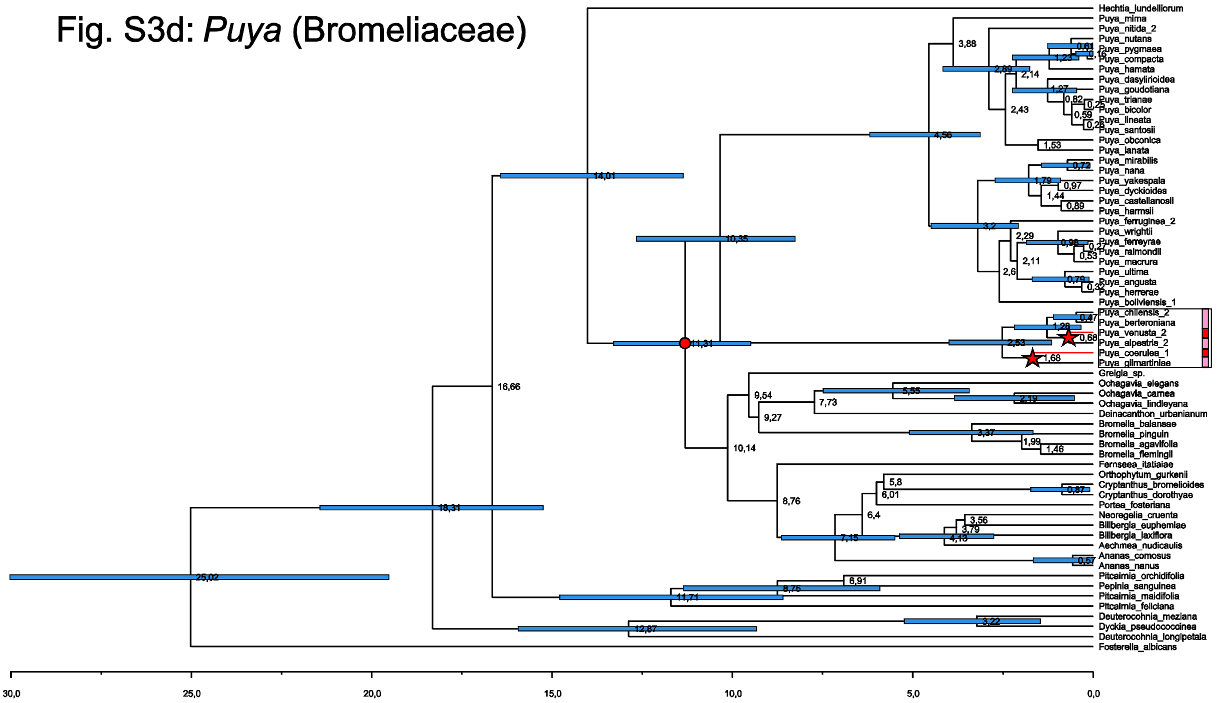

Fig. S3e: *Rhaphithamnus* (Verbenaceae)

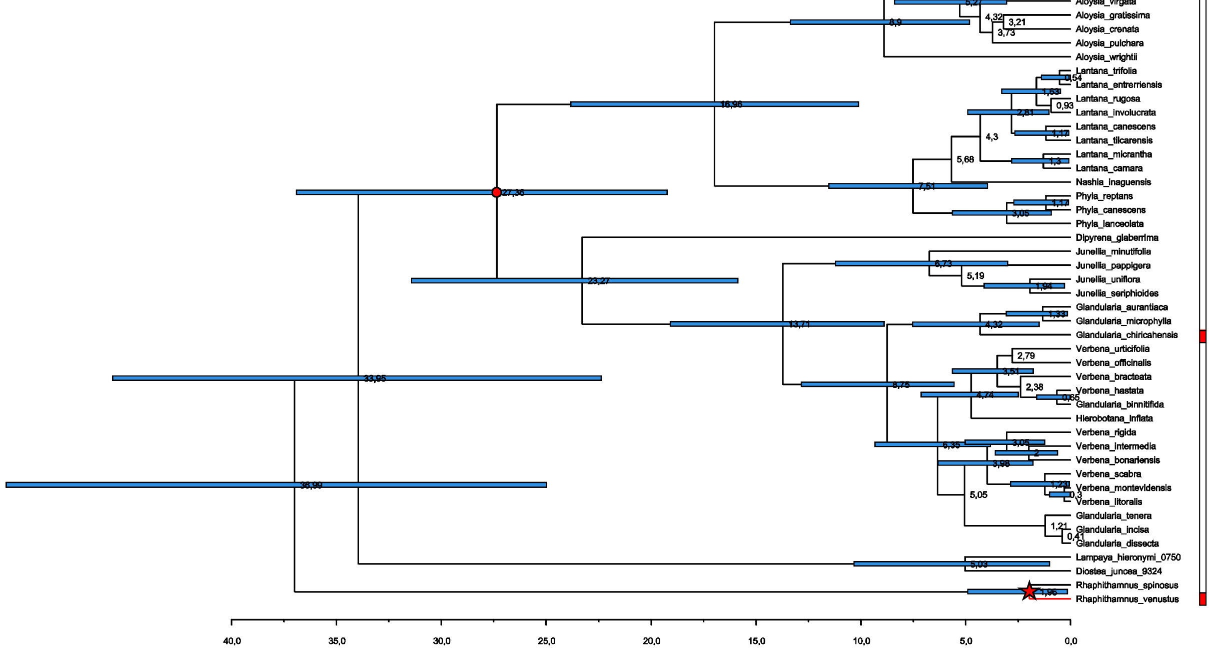

Fig. S3f: *Schizanthus* (Solanaceae)

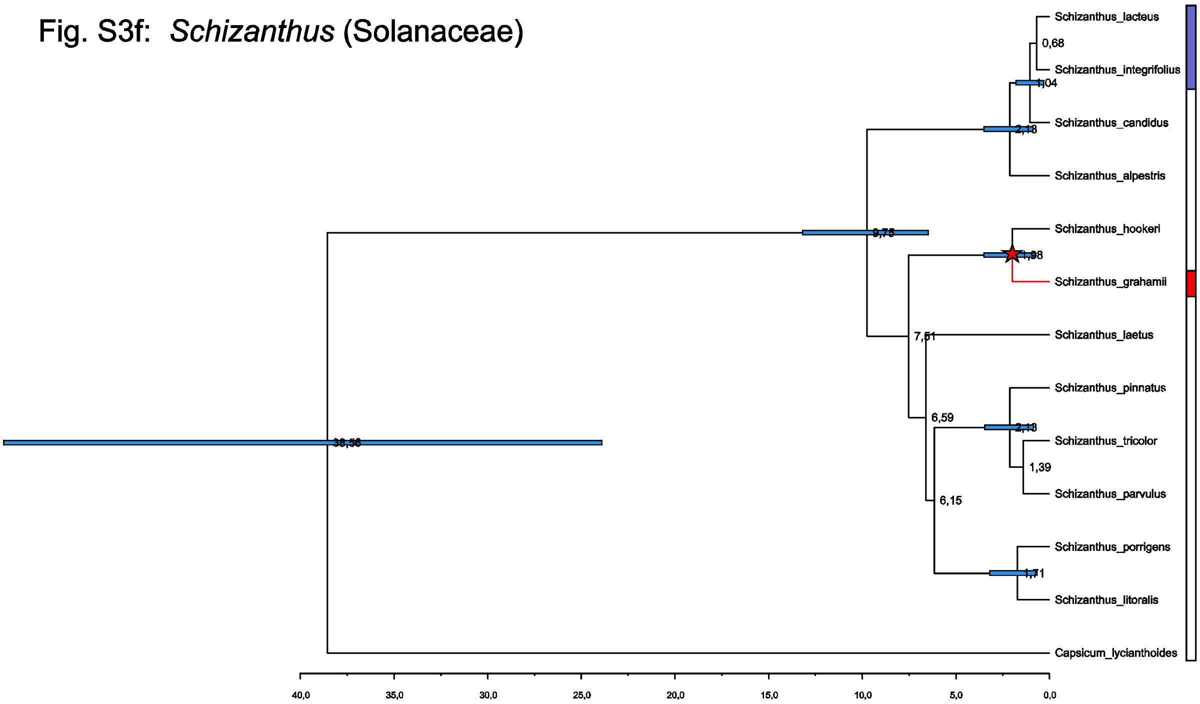

Fig. S3g: *Tristerix* (Loranthaceae)

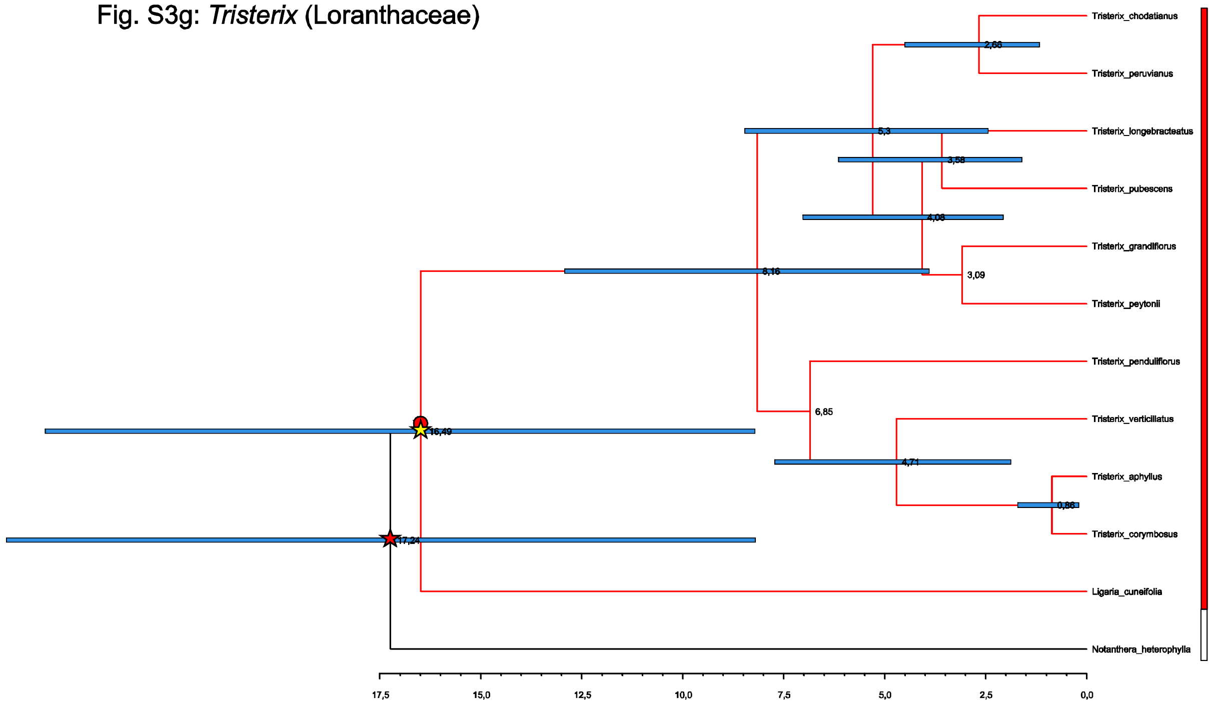

Fig. S3h *Vestia* (Solanaceae)

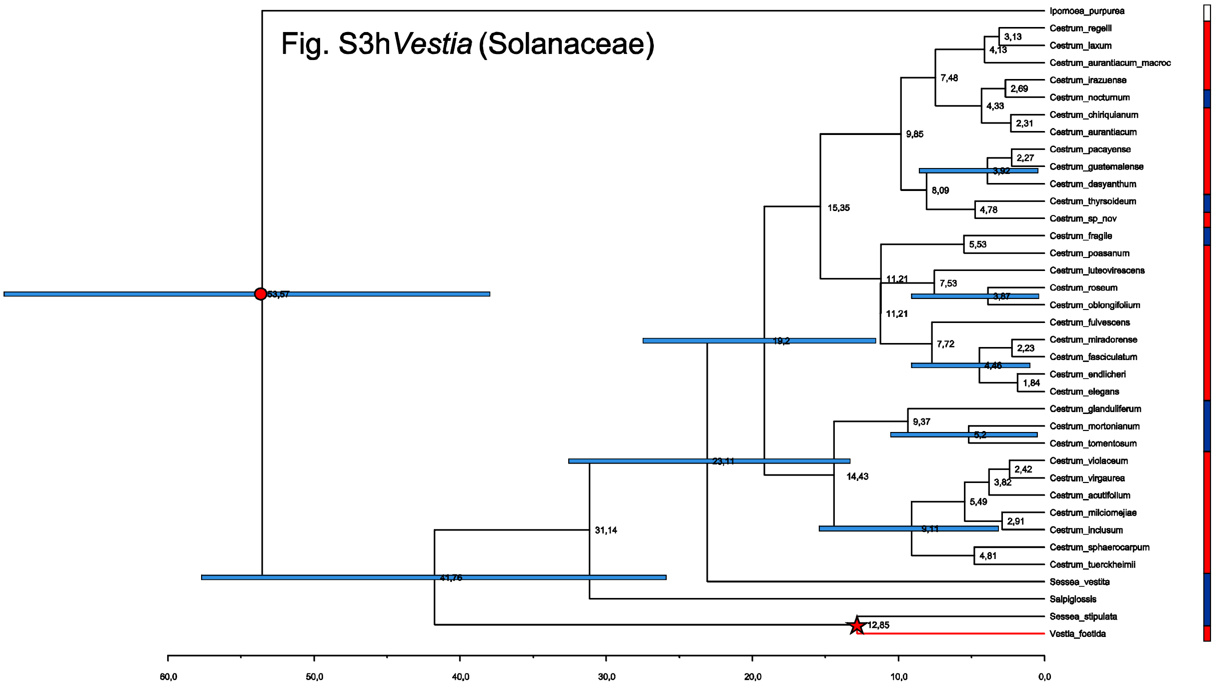

Supplement: Supplementary file 4 — Plant chronograms for temperate South American clades. [file 12862_2015_388_MOESM4_ESM.pdf]
