# Supplementary material for: The temporal build-up of hummingbird/plant mutualisms in North America and temperate South America
Source: BMC Evol Biol. 2015 Jun 10;15:104. doi: 10.1186/s12862-015-0388-z (PMC4460853; doi:10.1186/s12862-015-0388-z)
Supplement: Supplementary file 5 — Table S1 North American hummingbird species, with geographic ranges and divergence times from Fig. S1a. Node ages are followed by 95 % confidence intervals in brackets. Focal species in bold. Table S2. Temperate South American hummingbird species, with geographic ranges and divergence times from Fig. S1a. Node ages are followed by 95 % confidence intervals in brackets. Oreotrochilus leucopleurus has not been sequenced, and for this species we used the stem age of Oreotrochilus as the oldest possible age of the species, which could be much younger. Table S3 .Plant matrices newly clock-dated and/or used for ancestral state reconstructions for this study, 8 from North America and 8 from temperate South American; the GenBank accession numbers of a few sequences added to certain alignments (as specified in the online supporting material) are listed at the end of this table. Table S4. Hummingbird sequences from GenBank added to the alignment of McGuire et al. (2007). AK1 = intron 5 in the nuclear adenylate kinase (AK1) gene (ca. 660 base pairs [bp]); NADH subunit 4 and 2 = mitochondrial NADH dehydrogenase subunits 4 and 2 (ND4 and ND2, ca. 900 and ca. 1050 bp); Bfib = intron 7 in the beta fibrinogen (Bfib) gene (ca. 1100 bp). Table S5. Hummingbird-adapted plant species from North America. Ipomopsis aggregata subspecies are treated as separate species. Clades or species for which divergence times have been inferred (as cited in Table 1 and shown in the online chronograms) are marked in red. References for this table and :Table S6 are listed below S6. Table S6. Hummingbird-adapted plant species from temperate South America. Clades or species for which divergence times have been inferred (as cited in Table 2 and shown in the online chronograms) are marked in red. Table S7 .Sister taxa in which one member of a pair is pollinated by hummingbirds, the other is not, together with their species numbers. [file 12862_2015_388_MOESM5_ESM.doc]

**Online supporting material for** Abrahamczyk, S., and S. S. Renner: The temporal build-up of hummingbird/plant mutualisms in North America and temperate South America

**Table S1:** The North American hummingbird species, with their ages from the chronogram Fig. S1a and their geographic distribution. Node ages are followed by 95% confidence intervals in brackets. Focal species marked in bold.

| **Species** | **Group** | **Age fossil calibration**  **Fig. S1a** | **Age rate calibration**  **Fig. S1b** | **Age in McGuire *et al.* (2014)** | **Distribution** |
| --- | --- | --- | --- | --- | --- |
| ***Archilochus alexandri*** | Bee | 1.5  (1.07-1.94) | 1.58  (1.2-2.01) | 1.01  (0.69-1.36) | North America |
| ***Archilochus colubris*** | Bee | 1.5  (1.07-1.94) | 1.58  (1.2-2.01) | 1.01  (0.69-1.36) | North America |
| *Calothorax lucifer* | Bee | 2.24  (1.71-2.83) | 2.36  (1.83-2.86) | 1.85  (1.32-2.43) | Central America to  Southern Arizona |
| ***Calypte anna*** | Bee | 2.52  (2.01-3.13) | 2.66  (2.13-3.11) | 1.62  (1.23-2.03) | North America |
| ***Calypte costae*** | Bee | 2.52  (2.01-3.13) | 2.66  (2.13-3.11) | 1.62  (1.23-2.03) | North America |
| ***Selasphorus platycercus*** | Bee | 1.84  (1.41-2.26) | 1.93  (1.57-2.29) | 1.55  (1.05-1.61) | North America & Central America |
| ***Selasphorus rufus*** | Bee | 0.97  (0.7-1.24) | 1.02  (0.76-1.3) | 1.12  (0.88-1.38) | North America |
| ***Selasphorus sasin*** | Bee | 0.62  (0.29-0.7) | 0.65  (0.45-0.86) | 0.57  (0.44-0.73) | North America |
| ***Stellula calliope*** | Bee | 0.62  (0.29-0.7) | 0.65  (0.45-0.86) | 0.57  (0.44-0.73) | North America |
| *Amazilia beryllina* | Emerald | - | - | 0.34  (0.21-0.53) | Central America to  southern Arizona |
| *Amazilia violiceps* | Emerald | - | - | 0.17 (NA) | Central America to  southern Arizona |
| *Amazilia yucatanensis* | Emerald | - | - | 2.85  (2.14-3.58) | Central America to  southeastern Texas |
| *Cynanthus latirostris* | Emerald | - | - | 2.87  (2.25-3.66) | Central America to  southern Arizona |
| *Hylocharis leucotis* | Emerald | - | - | 2.57  (1.84-3.37) | Central America to  southern Arizona |
| *Hylocharis xantusii* | Emerald | - | - | 2.57  (1.84-3.37) | Southern part of  Baja California |
| *Eugenes fulgens* | Mountain Gem | 11.08  (9.5-12.8) | 11.67 (10.7-12.9) | 8.29  (6.92-9.56) | Central America to  southern Arizona,  New Mexico & Texas |
| *Heliomaster constantii* | Mountain Gem | - | - | 5.29  (4.43-6.28) | Central America to  northern Mexico |
| *Lampornis clemenciae* | Mountain Gem | - | - | 5.2  (4.39-6.21) | Central America to  southern Arizona  & Texas |

**Table S2:** The temperate South American hummingbird species, with their ages from the chronogram Fig. S1a and their geographic distribution. Node ages are followed by 95% confidence intervals in brackets. *Oreotrochilus leucopleurus* is not included in our tree, and for this species we used the stem age of *Oreotrochilus.*

| **Species** | **Group** | **Age fossil calibration**  **Fig. S1a** | **Age rate calibration**  **Fig. S1b** | **Age in McGuire *et al.* (2014)** | **Distribution** |
| --- | --- | --- | --- | --- | --- |
| *Sephanoides fernandensis* | Coquettes | 4.62  (3.63-5.65) | 4.84  (3.99-5.71) | 3.13  (2.43-3.98) | Temperate South  America |
| *Sephanoides sephaniodes* | Coquettes | 4.62  (3.63-5.65) | 4.84  (3.99-5.71) | 3.13  (2.43-3.98) | Temperate South  America |
| *Patagona gigas* | Patagoni | 15.8 (NA) | 17.36 (NA) | 14.44 (13.05-15.77) | Andes from southern  Colombia to central Chile |
| *Sappho sparganura* | Coquettes | 8.02  (6.69-9.38) | 8.43  (7.47-9.43) | 6.27  (5.3-7.44) | Southern Andes from  Bolivia to Argentina |
| *Oreotrochilus* (stem age) | Coquettes | 8.0 (NA) | 7.91 (NA) | 7.78  (6.86-8.76) | Southern Andes from  Bolivia to Central Chile |
| *Rhodopis vesper* | Bees | 2.23 (NA) | 2.36 (NA) | 1.45 (NA) | Central Andes from  Peru to Northern Chile |

**Table S3:** Plant matrices newly clock-dated and/or used for ancestral state reconstructions for this study, 8 from North America and 8 from temperate South American; the GenBank accession numbers of a few sequences added to certain alignments (as specified below) are listed at the end.

**North American clades**

***Aquilegia* (Ranunculaceae)**

Chronogram obtained by Bastida et al. (2010) under a Bayesian relaxed clock model applied to 34 accessions and 2486 aligned nucleotides of plastid and nuclear DNA sequences, using a fossil calibration. For the chronogram and ancestral state reconstruction see Figure S2a.

***Castilleja* (Orobanchaceae)**

We used the alignment of Tank and Olmstead (2008), which comprises 79 species of Castillejinae (Orobanchaceae), representing all major lineages, two outgroup species (*Paulownia tomentosa* (Paulowniaceae), *Rhinanthus alectorolophus* (Orobanchaceae)), and 3257 aligned positions of the nuclear ribosomal DNA internal transcribed spacers ITS1 and ITS2 and the intervening 5.8S gene, the external spacer ETS and the chloroplast rps16 and trnL-F regions. A strict clock model fit the data better than a relaxed clock (ucld.stdev: 0.353), and we calibrated it using a normally distributed secondary constraint of 38.0 ± 7.5 my for the split between Paulowniaceae and Orobanchaceae, based on the age (and error range) obtained by Bell *et al.* (2010) for this node. With this constraint, the age inferred for the split between Rhinantheae and its sister clade was 33.53 (25.45-41.8) my. This agrees with the age inferred by Gussarova *et al.* (2008) for the same split, 28.3 (21.18-30.59) my, even though their tree was incorrectly rooted. For the chronogram and ancestral state reconstruction see Figure S2b.

***Ipomopsis* and *Collomia* (Polemoniaceae)**

We used the alignment of Porter *et al.* (2010) comprising 100 taxa of Polemoniaceae (all 50 species of *Ipomopsis*) and 1234 aligned positions of the plastid *trnL* intron and *trnL-trnF* intergenic spacer. The nuclear ribosomal DNA internal transcribed spacers ITS1 and ITS2 and intervening 5.8S gene were omitted because of doubtfully aligned sections. A relaxed clock model fit the data better than a strict clock (ucld.mean 0.539), and we calibrated it using an average substitution rate for angiosperm plastid DNA of 1.3 substitutions/site/year x 10^-9^ from Richardson *et al.* (2001). We opted for this rate calibration instead of the *Gilisenium hueberi* fossil used by Porter et al. (2010) to constrain the stem of the tribe Gilieae to 98.0 my because the fossil-based dating yielded an extremely old age for Polemoniaceae compared to the age obtained for this family in the angiosperm-wide study of Bell *et al.* (2010). The node age inferred by us for the split between *Phlox* and the *Ipomopsis* clade, 25.84 (21.26-30.96) my, agrees with the age inferred by Bell *et al.* (2010) for the same node, 24.0 (12.0-37.0) my. For the chronogram see and ancestral state reconstruction Figure S2c.

***Keckiella* (Plantaginaceae)**

We modified the alignment of Wolfe *et al.* (2006), which comprises all seven species of *Keckiella* and ten outgroup species (Plantaginaceae), 1359 aligned positions of the nuclear ribosomal DNA internal transcribed spacers ITS1 and ITS2 and the intervening 5.8S gene and the trnL-F intergenic spacer, by adding sequences of *Keckiella antirrhinoides* and *Gambelia speciosa* from GenBank. A relaxed clock model fit the data better than a strict clock (ucld.stdev ITS: 0.585, ucld.stdev trnL-F 0.822), and we calibrated it using the ITS substitution rate of 4.72 substitutions/site/year x 10^-9^ from Kay *et al.* (2005) calculated for *Plantago*, a closely related genus. With this calibration, the age inferred for the split between *Antirrhinum* and *Gambelia* was 5.27 (1.81-9.05) my. This agrees with the age inferred by Vargas *et al.* (2009) for the same node, 7.25 (10.28-4.21) my. For the chronogram and ancestral state reconstruction see Figure S2d.

***Lithospermum* (Boraginaceae)**

Chronogram obtained by Cohen (2012) under a Bayesian relaxed clock model applied to 42 accessions and 10118 aligned nucleotides of plastid and nuclear DNA sequences, using a fossil calibration. For the chronogram and ancestral state reconstruction see Figure S2e.

***Lonicera* (Caprifoliaceae)**

Chronogram obtained by Smith and Donoghue (2010) under a Bayesian clock model applied to 17 accessions and about 1800 aligned nucleotides of plastid and nuclear DNA sequences, using a secondary calibration. For the chronogram and ancestral state reconstruction see Figure S2f.

***Monarda* (Lamiaceae)**

We downloaded ITS sequences from GenBank and constructed an alignment (available under www.treebase.org: No. 14591 NOT FOUND) in Mesquite (*Materials and Methods*), which comprised 14 out of 16 known *Monarda* species, five outgroup species (4 Lamiaceae and 1 Orobanchaceae), and 970 aligned positions of the nuclear ribosomal DNA internal transcribed spacers ITS1 and ITS2, together with the intervening 5.8S gene. A relaxed clock model fit the data better than a strict clock because the ucld.stdev value was 1.21, and we calibrated it with an intermediate ITS substitution rate of 1.99 substitutions/site/year x 10^-9^ from Kay et al. (2006) because perennial herbs, such as *Monarda* have been found to have medium ITS substitution rates (Soria-Hernanz *et al.*, 2008). With this calibration, the age inferred for the split between Lamiaceae and their sister clade, containing Orobanchaceae and Paulowniaceae, was 46.76 (20.58-83.68) my. This agrees with the age inferred by Bell *et al.* (2010) for the same split, 48.0 (39.0-56.0) my. For the resulting chronogram and ancestral state reconstruction see Figure S2g.

***Ribes* (Grossulariaceae)**

We used the alignment of Schultheis and Donoghue (2004), which comprises 49 of the 150 species of *Ribes* and two species of *Itea*, and has 1259 aligned positions of the nuclear ribosomal DNA external spacer (ETS), the internal transcribed spacers ITS1 and ITS2, and the intervening 5.8S gene. As an additional outgroup we added a sequence of *Mitella* (Saxifragaceae) from GenBank. We used a relaxed clock (ETS ucld.stdev: 1.45, ITS ucld.stdev: 0.85) and calibrated it using an ITS substitution rate of 1.2 substitutions/site/year x 10^-9^. This rate lies between the slow rates of trees, such as *Alnus* (Betulaceae; 1.1 substitutions/site/year x 10^-9^), and the medium rates of dwarf shrubs, such as *Empetrum* (Ericaceae; 1.44 substitutions/site/year x 10^-9^) (Kay *et al.*, 2005). The age inferred with this rate for the split between Saxifragaceae and Iteaceae was 71.09 (35.54-111.09) my, which agrees with the age of 77.0 (60.0-88.0) my inferred by Bell *et al.* (2010) for the same node. The resulting chronogram and ancestral state reconstruction is shown as Figure S2h.

**South American clades**

***Campsidium* (Bignoniaceae)**

We slightly modified the alignment of Olmstead *et al.* (2009), which comprises 86 species of Bignoniaceae, representing all major clades and most genera, two outgroup species (Verbenaceae), and 3327 aligned positions of the plastid genes *rbcL* and *ndhF* and plastid spacer *trnL-trnF*. A strict clock model fit the data better than a relaxed clock (relaxed clock ucld.stdev: 0.47), and we calibrated it using a plastid rate of 0.995 substitutions/site/year x 10^-9^ from Särkinen *et al.* (2007). With this calibration, the age inferred for the split between Tecomeae and its sister clade (several Bignoniaceae subfamilies) was 26.1 (22.1-30.1) my. This agrees with the age inferred by Bell *et al.* (2010) for the same node, 25.0 (18.0-31.0) my. For the chronogram and ancestral state reconstruction see Figure S3a.

***Dendroseris* (Asteraceae)**

We used the plastid alignment of Kim *et al.* (2007), which comprises five of the eleven *Dendroseris* species, 60 other Sonchinae species (Asteraceae), and 580 aligned positions of the nuclear ribosomal DNA internal transcribed spacers ITS1 and ITS2 and the intervening 5.8S gene. As an additional outgroup we added a sequence of *Helianthus annuus* (Asteraceae) from GenBank. A relaxed clock model fit the data better than a strict clock (ucld.stdev: 1.34), and we calibrated it with an ITS substitution rate of 6.0 substitutions/site/year x 10^-9^ calculated by Richardson *et al.* (2008) for *Dendroseris*. With this calibration, the age inferred for the split between Cichorieae and Heliantheae was 27.33 (18.56-37.98) my. This agrees with the age of 27.0 (19.0-35.0) my inferred by Bell *et al.* (2010) for the same node. For the chronogram and ancestral state reconstruction see Figure S3b.

***Latua* (Solanaceae)**

We modified the alignment of Olmstead *et al.* (2008), which comprises all major lineages and most genera of Solanaceae (183 species) and 3040 aligned positions of the plastid *ndhF* and *trnL-trnF* regions. A relaxed clock model fit the data better than a strict clock (ucld.stdev: 0.521), and we calibrated it with a normally distributed secondary constraint of 38.0 (29.0-47.0) my for the crown group of Solanaceae (Bell *et al.*, 2010). MCMC chains were run for 40 million generations, sampling every 10,000th generation to reach stationarity in Tracer (*Materials and Methods*). The age inferred with this calibration for the *Petunia* clade was 9.63 (4.63-16.19) my. This agrees with the age of 12.0 my inferred for the same clade by Filipowicz and Renner (2012). For the chronogram and ancestral state reconstruction see Figure S3c.

***Puya* (Bromeliaceae)**

We used the alignment of Jabaily and Sytsma (2010), which comprises 35 out of approximately 190 species of *Puya* including all basal species, 29 outgroup species (Bromeliaceae), and 2553 aligned positions of the plastid gene *matK*, the intergenic spacer *trnS-trnG,* and the *rps16* intron, and the nuclear gene *PHYC.* A strict clock model fit the data better than a relaxed clock (ucld.stdev relaxed clock: 0.4), and because of the large plastid component of this matrix we calibrated the clock model with a plastid substitution rate of 4.87 substitutions/site/year x 10^-10^ from Richardson *et al.* (2001). With this calibration, the age inferred for the split between *Puya* and the Bromelioideae clade was 11.31 (9.46-13.28) my. This agrees with the age of 14.0 (7.0-22.0) my inferred by Bell *et al.* (2010) for the same node. For the chronogram and ancestral state reconstruction see Figure S3d.

***Rhaphithamnus* (Verbenaceae)**

We used a reduced alignment of Marx *et al.* (2010), which comprises 46 species and most lineages of Verbenaceae, including both *Rhaphithamnus* species and 3065 aligned positions of the plastid genes *ndhF*, *matK*, *rbcL*, *rpoC2*, *ccsA* and *rps3* and the spacer *trnL-trnF*. A relaxed clock model fit the data better than a strict clock (ucld.stdev: 0.774), and we calibrated it using an average plastid substitution rate of 6.0 substitutions/site/year x 10^-10^ calculated for the likewise woody genus *Gleditsia* (Fabaceae) by Schnabel and Wendel (1998). MCMC chains were run for 32 million generations, sampling every 10,000th generation to reach stationarity in Tracer. The age inferred for the split between Lantaneae and Verbeneae was 27.36 (19.12-36.87) my, which agrees with the age of 29.0 (18.0-39.0) my inferred by Bell *et al.* (2010) for the same node. For the chronogram and ancestral state reconstruction see Figure S3e.

***Schizanthus* (Solanaceae)**

We modified the alignment of Pérez *et al.* (2006), which comprises all 12 *Schizanthus* species and 1566 aligned positions of the nuclear ribosomal DNA internal transcribed spacers ITS1 and ITS2, the intervening 5.8S gene, and the *Waxy* gene. As an additional outgroup we added GenBank sequences of *Capsicum lycianthoides*. A relaxed clock model fit the data better than a strict clock (ucld.stdev ITS: 0.226; *Waxy* 0.587), and we calibrated it with an ITS substitution rate from the biannual Rhamnaceae *Phylica* of 2.44 substitutions/site/year x 10^-10^ from Kay *et al.* (2006). The *Waxy* region was run without a rate prior. The age inferred with this calibration for the split between *Schizanthus* and *Capsicum* was 38.56 my. This agrees with the age inferred by Bell *et al.* (2010), 37.0 (29.0-47.0) my, for the same node. For the chronogram and ancestral state reconstruction see Figure S3f.

***Tristerix* (Loranthaceae)**

We used the alignment of Amico *et al.* (2007), which comprises 11 out of 13 species of *Tristerix* and *Ligaria*, one outgroup species (Loranthaceae), and 2176 aligned positions of the nuclear ribosomal DNA internal transcribed spacers ITS1 and ITS2 and the intervening 5.8S gene, and the plastid DNA spacers *trnL-trnF* and *atpB-rbcL*. A relaxed clock model fit the data better than a strict clock (ucld.stdev: 0.994), and we calibrated it using an ITS substitution rate of 7.0 substitutions/site/year x 10^-9^, which is intermediate between the rates of woody *Dendroseris* (Asteraceae) and semi-woody *Gossypium* (Malvaceae). We choose this rate because *Tristerix* is a woody, epiphytic parasite. The plastid regions were run without a fixed rate prior. With this calibration, the age inferred for the split between *Tristerix* and *Ligaria* was 16.5 (8.23-25.68) my. This agrees with the age of 17.4 my inferred by Vidal-Russell and Nickrent (2008) for the same node. For the chronogram and ancestral state reconstruction see Figure S3g.

***Vestia* (Solanaceae)**

We used the plastid alignment of Montero-Castro *et al.* (2006), which comprises 32 out of 175 *Cestrum* species, five outgroup species (4 Solanaceae), and 3492 aligned positions of the plastid gene *matK* and the *trnL-trnF* and *trnT-trnL* spacers. We added *Ipomoea purpurea* (Convolvulaceae) as an additional outgroup. A relaxed clock model fit the data better than a strict clock (ucld.stdev: 1.34), and we calibrated it with an average plastid substitution rate of 7.0 substitutions/site/year x 10^-10^ from Palmer (1991). With this calibration, the age inferred for the split between Convolvulaceae and Solanaceae was 53.57 (37.95-71.15) my. This agrees with the age inferred by Bell *et al.* (2010) for the same node, 59.0 (49.0-68.0) my. For the chronogram and ancestral state reconstruction see Figure S3h.

**GenBank accession numbers for sequences added to the above-cited alignments.**

|  | ***trnL-trnF*** | **ITS** | ***rps16*** | ***trnT-trnL*** | **ETS** |
| --- | --- | --- | --- | --- | --- |
| *Mitella furusei*  (Saxifragaceae) |  | AF158959 |  |  | AB248775 |
| *Rhinanthus alectorolophus*  (Orobanchaceae) | JQ910137 | JF900501 | JF900534 | EU264173 |  |
| *Paulownia tomentosa*  (Paulowniaceae) | AF479005 | AF478941 | HQ385149 |  | AF478974 |
| *Pedicularis vialii*  (Orobanchaceae) |  | FJ792969 |  |  |  |
| *Lamium amplexicaule*  (Lamiaceae) |  | AB266245 |  |  |  |
| *Callicarpa dichotoma*  (Lamiaceae) |  | AF478942 |  |  |  |
| *Clerodendrum paniculatum*  (Lamiaceae) |  | U77765 |  |  |  |
| *Mentha Canadensis*  (Lamiaceae) |  | JN407476 |  |  |  |
| *Monarda austromontana*  (Lamiaceae) |  | AY771705 |  |  |  |
| *Monarda bartlettii* |  | AF369191 |  |  |  |
| *Monarda bradburiana* |  | GU381418 |  |  |  |
| *Monarda citriodora* |  | JQ669124 |  |  |  |
| *Monarda clinopodia* |  | AF369190 |  |  |  |
| *Monarda didyma* |  | AF369194 |  |  |  |
| *Monarda eplingiana* |  | AF369196 |  |  |  |
| *Monarda fistulosa* |  | DQ667318 |  |  |  |
| *Monarda lindheimeri* |  | AF369206 |  |  |  |
| *Monarda pectinata* |  | AF369174 |  |  |  |
| *Monarda pringlei* |  | AF369208 |  |  |  |
| *Monarda punctata* |  | AF369184 |  |  |  |
| *Monarda russeliana* |  | AF369188 |  |  |  |
| *Monarda stipitatoglandulosa* |  | AF369210 |  |  |  |
| *Monarda viridissima* |  | AF369180 |  |  |  |
| *Keckiella antirrhinoides*  (Plantaginaceae) |  | AF343541 |  |  |  |
| *Gambelia speciosa*  (Plantaginaceae) |  | AY880224 |  |  |  |

**Table S4:** Hummingbird sequences from GenBank added to the alignment of McGuire et al. (2007). AK1 = intron 5 in the nuclear *adenylate kinase* (AK1) gene (ca. 660 base pairs [bp]); NADH subunit 4 and 2 = mitochondrial *NADH dehydrogenase* subunits 4 and 2 (ND4 and ND2, ca. 900 and ca.1050 bp); Bfib = intron 7 in the *beta fibrinogen* (Bfib) gene (ca. 1100 bp).

|  | **AK1** | **NADH subunit 4** | **NADH subunit 2** | **Bfib** |
| --- | --- | --- | --- | --- |
| *Aglaeactis pamela* | GU167147 | GU166869 | GU167201 | GU167091 |
| *Amazilia amazilia* | GU167148 | GU166870 | GU167202 | GU167092 |
| *Amazilia lactea* | GU167149 | GU166871 | GU167203 | GU167093 |
| *Amazilia rosenbergi* | GU167150 | GU166872 | GU167204 | GU167094 |
| *Amazilia viridicauda* | GU167152 | GU166874 | GU167206 | GU167096 |
| *Anthocephala floriceps* | GU167154 | GU166876 | GU167208 | GU167098 |
| *Anthracothorax prevostii* | GU167155 | GU166877 | GU167209 | GU167099 |
| *Atthis heliosa* | KJ601816 | KJ602393 | KJ602190 | KJ602004 |
| *Avocettula recurvirostris* | GU167156 | GU166878 | GU167210 | GU167100 |
| *Calliphlox amethystina* | GU167157 | GU166879 | GU167211 | GU167101 |
| *Calliphlox evelynae* | KJ601817 | KJ602396 | KJ602192 | KJ602006 |
| *Calothorax lucifer* | KJ601819 | KJ602397 | KJ602194 | KJ602008 |
| *Campylopterus cuvierii* | GU167190 | GU166913 | GU167244 | GU167134 |
| *Campylopterus falcatus* | GU167158 | GU166881 | GU167212 | GU167102 |
| *Chaetocercus bombus* | GU167159 | GU166882 | GU167213 | GU167103 |
| *Chalcostimga olivaceum* | GU166828 | GU166858 | GU166848 | GU166839 |
| *Chlorostilbon aureoventris* | GU167160 | GU166883 | GU167214 | GU167104 |
| *Chlorostilbon poortmani* | GU167161 | GU166884 | GU167215 | GU167105 |
| *Coeligena bonapartei* | GU167162 | GU166885 | GU167216 | GU167106 |
| *Coeligena helianthea* | FJ903654 | FJ903618 | FJ903510 | FJ903582 |
| *Coeligena iris* | GU167164 | GU166887 | GU167218 | GU167108 |
| *Coeligena orina* | GU167165 | GU166888 | GU167219 | GU167109 |
| *Coeligena phalerata* | GU167166 | GU166889 | GU167220 | GU167110 |
| *Coeligena prunellei* | GU167167 | GU166890 | GU167221 | GU167111 |
| *Colibri serrirostris* | GU167168 | GU166891 | GU167222 | GU167112 |
| *Discosura langsdorffi* | GU167169 | GU166892 | GU167223 | GU167113 |
| *Discosura longicauda* | GU167170 | GU166893 | GU167224 | GU167114 |
| *Doricha elizae* | KJ601861 | KJ602438 | KJ602238 | KJ602047 |
| *Eriocnemis cupreoventris* | GU167171 | GU166894 | GU167225 | GU167115 |
| *Eriocnemis glaucopoides* | GU167172 | GU166895 | GU167226 | GU167116 |
| *Eriocnemis nigrivestis* | GU167173 | GU166896 | GU167227 | GU167117 |
| *Eupetomena macroura* | GU167174 | GU166897 | GU167228 | GU167118 |
| *Florisuga fusca* | GU167175 | GU166898 | GU167229 | GU167119 |
| *Heliangelus micraster* | GU167176 | GU166899 | GU167230 | GU167120 |
| *Heliangelus micraster* | GU167176 | GU166899 | GU167230 | GU167120 |
| *Heliangelus regalis* | GU166832 | GU166860 | GU166850 | GU166841 |
| *Heliangelus strophianus* | GU167177 | GU166900 | GU167231 | GU167121 |
| *Heliangelus viola* | GU167178 | GU166901 | GU167232 | GU167122 |
| *Heliodoxa gularis* | GU167179 | GU166902 | GU167233 | GU167123 |
| *Heliomaster furcifer* | GU167180 | GU166903 | GU167234 | GU167124 |
| *Heliothryx auritus* | GU167181 | GU166904 | GU167235 | GU167125 |
| *Hylocharis chrysura* | GU167182 | GU166905 | GU167236 | GU167126 |
| *Lampornis calolaemus* | EU042480 | EU042245 | GU167239 | EU042400 |
| *Leucippus baeri* | GU167183 | GU166906 | GU167237 | GU167127 |
| *Leucippus chlorocercus* | GU167184 | GU166907 | GU167238 | GU167128 |
| *Lophornis chalybeus* | GU167185 | GU166908 | GU167239 | GU167129 |
| *Mellisuga minima* | KJ601891 | KJ602470 | KJ602269 | KJ602075 |
| *Metallura baroni* | GU167186 | GU166909 | GU167240 | GU167130 |
| *Metallura eupogon* | GU166833 | GU166863 | GU166852 | GU166842 |
| *Metallura odomae* | GU167187 | GU166910 | GU167241 | GU167131 |
| *Metallura theresiae* | GU166834 | GU166864 | AF022681 | GU166843 |
| *Microstilbon burmeisteri* | GU167188 | GU166911 | GU167242 | GU167132 |
| *Myrmia micrura* | GU167189 | GU166912 | GU167243 | GU167133 |
| *Oreotrochilus melanogaster* | GU166835 | GU166866 | GU166854 | GU166844 |
| *Phaethornis eurynome* | GU167191 | GU166914 | GU167245 | GU167135 |
| *Phaethornis pretrei* | GU167192 | GU166916 | GU167247 | GU167136 |
| *Phaethornis subochraceus* | GU167193 | GU166917 | GU167248 | GU167137 |
| *Polyonymus caroli* | GU166836 | GU166867 | GU166855 | GU166845 |
| *Sappho sparganura* | GU167194 | GU166918 | GU167249 | GU167138 |
| *Selasphorus ardens* | KJ601930 | KJ602509 | KJ602311 | KJ602109 |
| *Selasphorus sasin* | KJ601947 | KJ602529 | KJ602331 | KJ602128 |
| *Selasphorus scintilla* | KJ601948 | KJ602531 | KJ602333 | KJ602129 |
| *Stephanoxis lalandi* | GU167195 | GU166919 | GU167250 | GU167139 |
| *Taphrolesbia griseiventris* | GU166837 | GU166868 | GU166856 | GU166846 |
| *Thalurania glaucopis* | GU167196 | GU166920 | GU167251 | GU167140 |
| *Thaumastura cora* | GU167197 | GU166921 | GU167252 | GU167141 |
| *Urosticte ruficrissa* | GU167199 | GU166925 | GU167256 | GU167145 |

**Table S5:** Hummingbird-adapted plant species of North America. Note that *Ipomopsis aggregata* subspecies are treated as separate species. Clades or species for which divergence times have been inferred (as cited in Table 1) are marked in red. References for this table and Table S6 are listed below S6.

| **Species** | **Family** | **Reference** |
| --- | --- | --- |
| *Aesculus pavia* | Sapindaceae | Bertin, 1982 |
| *Agastache rupestris* | Lamiaceae | McDonald, 2013 |
| *Anisacanthus andersonii* | Acanthaceae | Hagen, 1941 |
| *Anisacanthus linearis* | Acanthaceae | Hagen, 1941 |
| *Anisacanthus puberulus* | Acanthaceae | Hagen, 1941 |
| *Anisacanthus quadrifidus* | Acanthaceae | Hagen, 1941 |
| *Anisacanthus thurberi* | Acanthaceae | Grant, 1994 |
| *Aquilegia canadensis* | Ranunculaceae | Bastida *et al*., 2010 |
| *Aquilegia desertorum* | Ranunculaceae | This study |
| *Aquilegia elegantula* | Ranunculaceae | Bastida *et al*., 2010 |
| *Aquilegia eximia* | Ranunculaceae | Grant, 1994 |
| *Aquilegia flavescens* | Ranunculaceae | Bastida *et al*., 2010 |
| *Aquilegia formosa* | Ranunculaceae | Bastida *et al*., 2010 |
| *Aquilegia shockleyi* | Ranunculaceae | Grant, 1994 |
| *Aquilegia skinneri* | Ranunculaceae | Bastida *et al*., 2010 |
| *Aquilegia triternata* | Ranunculaceae | Grant, 1994 |
| *Arbutus peninsularis* | Ericaceae | Arriaga *et al.*, 1990 |
| *Astragalus coccineus* | Fabaceae | Grant, 1994 |
| *Behria tenuiflora* | Asparagaceae | Arriaga *et al.*, 1990 |
| *Bignonia capreolata* | Bignoniaceae | This study |
| *Bouvardia glaberrima* | Rubiaceae | Grant, 1994 |
| *Calliandra peninsularis* | Fabaceae | Arriaga *et al.*, 1990 |
| *Campsis radicans* | Bignoniaceae | Bertin, 1982 |
| *Castilleja affinis* | Orobanchaceae | Grant, 1994 |
| *Castilleja angustifolia* | Orobanchaceae | Grant, 1994 |
| *Castilleja applegatei* | Orobanchaceae | Grant, 1994 |
| *Castilleja austromontana* | Orobanchaceae | Grant, 1994 |
| *Castilleja brevilobata* | Orobanchaceae | Grant, 1994 |
| *Castilleja breweri* | Orobanchaceae | Grant, 1994 |
| *Castilleja bryantii* | Orobanchaceae | Arriaga *et al.*, 1990 |
| *Castilleja chromosa* | Orobanchaceae | Grant, 1994 |
| *Castilleja coccinea* | Orobanchaceae | Duffield, 1971 |
| *Castilleja covilleana* | Orobanchaceae | Grant, 1994 |
| *Castilleja crista-galli* | Orobanchaceae | Grant, 1994 |
| *Castilleja cruenta* | Orobanchaceae | Grant, 1994 |
| *Castilleja elata* | Orobanchaceae | Grant, 1994 |
| *Castilleja elmeri* | Orobanchaceae | Grant, 1994 |
| *Castilleja exilis* | Orobanchaceae | Grant, 1994 |
| *Castilleja foliolosa* | Orobanchaceae | Grant, 1994 |
| *Castilleja francisana* | Orobanchaceae | Grant, 1994 |
| *Castilleja fraterna* | Orobanchaceae | Grant, 1994 |
| *Castilleja hispida* | Orobanchaceae | Grant, 1994 |
| *Castilleja inconstans* | Orobanchaceae | Grant, 1994 |
| *Castilleja indivisa* | Orobanchaceae | Bertin, 1982 |
| *Castilleja integra* | Orobanchaceae | Grant, 1994 |
| *Castilleja integrifolia* | Orobanchaceae | Grant, 1994 |
| *Castilleja lanata* | Orobanchaceae | Grant, 1994 |
| *Castilleja latifolia* | Orobanchaceae | Grant, 1994 |
| *Castilleja laxa* | Orobanchaceae | Grant, 1994 |
| *Castilleja leschkeana* | Orobanchaceae | Grant, 1994 |
| *Castilleja linariifolia* | Orobanchaceae | Grant, 1994 |
| *Castilleja martinii* | Orobanchaceae | Grant, 1994 |
| *Castilleja miniata* | Orobanchaceae | Grant, 1994 |
| *Castilleja minor* | Orobanchaceae | Grant, 1994 |
| *Castilleja oreopola* | Orobanchaceae | Duffield, 1971 |
| *Castilleja organorum* | Orobanchaceae | Grant, 1994 |
| *Castilleja patriotica* | Orobanchaceae | Grant, 1994 |
| *Castilleja peckiana* | Orobanchaceae | This study |
| *Castilleja peirsonii* | Orobanchaceae | Grant, 1994 |
| *Castilleja praeterita* | Orobanchaceae | This study |
| *Castilleja pruinosa* | Orobanchaceae | Grant, 1994 |
| *Castilleja rhexifolia* | Orobanchaceae | Grant, 1994 |
| *Castilleja roseana* | Orobanchaceae | Grant, 1994 |
| *Castilleja rupicola* | Orobanchaceae | Grant, 1994 |
| *Castilleja stenantha* | Orobanchaceae | Grant, 1994 |
| *Castilleja subinclusa* | Orobanchaceae | Grant, 1994 |
| *Castilleja suksdorfii* | Orobanchaceae | Grant, 1994 |
| *Castilleja tenuiflora* | Orobanchaceae | This study |
| *Castilleja wightii* | Orobanchaceae | Grant, 1994 |
| *Clinopodium coccineum* | Lamiaceae | Bertin, 1982 |
| *Collomia rawsoniana* | Polemoniaceae | Grant, 1994 |
| *Delphinium cardinale* | Ranunculaceae | Grant, 1994 |
| *Delphinium nudicaule* | Ranunculaceae | Grant, 1994 |
| *Dichelostemma ida-maia* | Asparagaceae | Grant, 1994 |
| *Dichelostemma venusta* | Asparagaceae | Grant, 1994 |
| *Echinocereus triglochidiatus* | Cactaceae | Grant, 1994 |
| *Epilobium californica* | Onagraceae | Grant, 1994 |
| *Epilobium cana* | Onagraceae | Grant, 1994 |
| *Epilobium garrettii* | Onagraceae | Grant, 1994 |
| *Epilobium septentriolaris* | Onagraceae | Grant, 1994 |
| *Erythrina coralloides* | Fabaceae | Steiner, 1979 |
| *Erythrina herbacea* | Fabaceae | Steiner, 1979 |
| *Eucnide aurea* | Loasaceae | This study |
| *Eucnide cordata* | Loasaceae | This study |
| *Fouquieria splendens* | Fouquieriaceae | Grant, 1994 |
| *Fritillaria recurva* | Liliaceae | Grant, 1994 |
| *Galvezia speciosa* | Plantaginaceae | Grant, 1994 |
| *Gilia subnuda* | Polemoniaceae | Grant, 1994 |
| *Hedeoma ciliosa* | Lamiaceae | This study |
| *Hedeoma todsenli* | Lamiaceae | Ulaszek, 2001 |
| *Hesperaloe engelmannii* | Asparagaceae | This study |
| *Hesperaloe parviflora* | Asparagaceae | Pellmyr & Augenstein, 1997 |
| *Impatiens capensis* | Balsaminaceae | Bertin, 1982 |
| *Ipomoea coccinea* | Convolvulaceae | Grant, 1994 |
| *Ipomopsis aggregata ssp. aggregata* | Polemoniaceae | Grant, 1994 |
| *Ipomopsis aggregata ssp. attenuata* | Polemoniaceae | This study |
| *Ipomopsis aggregata ssp. bridgesii* | Polemoniaceae | This study |
| *Ipomopsis aggregata ssp. collina* | Polemoniaceae | This study |
| *Ipomopsis aggregata ssp. formosissima* | Polemoniaceae | This study |
| *Ipomopsis arizonica* | Polemoniaceae | Grant, 1994 |
| *Ipomopsis rubra* | Polemoniaceae | Estes & Halli, 1975 |
| *Ipomopsis sancti-spiritus* | Polemoniaceae | Grant, 1994 |
| *Ipomopsis tenuifolia* | Polemoniaceae | Grant, 1994 |
| *Iris nelsonii* | Iridaceae | Taylor *et al.*, 2012 |
| *Justicia californica* | Acanthaceae | Grant, 1994 |
| *Justicia candicans* | Acanthaceae | Grant, 1994 |
| *Keckiella cordifolia* | Plantaginaceae | Grant, 1994 |
| *Keckiella corymbosa* | Plantaginaceae | Grant, 1994 |
| *Keckiella ternata* | Plantaginaceae | Grant, 1994 |
| *Lepechinia hastata* | Lamiaceae | Arriaga *et al.*, 1990 |
| *Lilium maritimum* | Liliaceae | Grant, 1994 |
| *Lithispermum flavum* | Boraginaceae | This study |
| *Lithospermum johnstonii* | Boraginaceae | This study |
| *Lithospermum leonotis* | Boraginaceae | This study |
| *Lithospermum macromeria* | Boraginaceae | Grant, 1994 |
| *Lithospermum notatum* | Boraginaceae | This study |
| *Lobelia cardinalis* | Campanulaceae | Grant, 1994 |
| *Lobelia laxiflora* | Campanulaceae | Grant, 1994 |
| *Loeselia mexicana* | Polemoniaceae | Abrol, 2012 |
| *Lonicera arizonica* | Caprifoliaceae | Grant, 1994 |
| *Lonicera ciliosa* | Caprifoliaceae | Grant, 1994 |
| *Lonicera dioica* | Caprifoliaceae | This study |
| *Lonicera involucrata var. ledebourii* | Caprifoliaceae | Grant, 1994 |
| *Lonicera sempervirens* | Caprifoliaceae | Williamson, 2001 |
| *Macranthera flammea* | Orobanchaceae | Alford & Anderson, 2002 |
| *Mimulus aurantiacus* | Phrymaceae | Grant, 1994 |
| *Mimulus cardinalis* | Phrymaceae | Grant, 1994 |
| *Mimulus eastwoodiae* | Phrymaceae | Grant, 1994 |
| *Mimulus longiflorus* | Phrymaceae | Grant, 1994 |
| *Mimulus parviflorus* | Phrymaceae | Grant, 1994 |
| *Mimulus puniceus* | Phrymaceae | Grant, 1994 |
| *Mirabilis coccinea* | Nyctaginaceae | Grant, 1994 |
| *Monarda didyma* | Lamiaceae | Whitten, 1981 |
| *Monardella macrantha* | Lamiaceae | This study |
| *Pedicularis densiflora* | Orobanchaceae | Grant, 1994 |
| *Penstemon alamosensis* | Plantaginaceae | Wolfe *et al.*, 2006 |
| *Penstemon baccharifolius* | Plantaginaceae | Wolfe *et al.*, 2006 |
| *Penstemon barbatus* | Plantaginaceae | Grant, 1994 |
| *Penstemon bridgesii* | Plantaginaceae | Grant, 1994 |
| *Penstemon cardinalis* | Plantaginaceae | Grant, 1994 |
| *Penstemon centranthifolius* | Plantaginaceae | Grant, 1994 |
| *Penstemon clevelandii* | Plantaginaceae | Grant, 1994 |
| *Penstemon crassulus* | Plantaginaceae | Grant, 1994 |
| *Penstemon eatonii* | Plantaginaceae | Grant, 1994 |
| *Penstemon fasciculatus* | Plantaginaceae | Wolfe *et al*., 2006 |
| *Penstemon havardii* | Plantaginaceae | Wolfe *et al.*, 2006 |
| *Penstemon isophyllus* | Plantaginaceae | Wolfe *et al*., 2006 |
| *Penstemon kunthii* | Plantaginaceae | Wolfe *et al*., 2006 |
| *Penstemon labrosus* | Plantaginaceae | Grant, 1994 |
| *Penstemon labrosus* | Plantaginaceae | Wolfe *et al.*, 2006 |
| *Penstemon lanceolatus* | Plantaginaceae | Wolfe *et al*., 2006 |
| *Penstemon newberryi* | Plantaginaceae | Grant, 1994 |
| *Penstemon parryi* | Plantaginaceae | Grant, 1994 |
| *Penstemon pinifolius* | Plantaginaceae | Grant, 1994 |
| *Penstemon racemosus* | Plantaginaceae | Wolfe *et al*., 2006 |
| *Penstemon rostriflorus* | Plantaginaceae | Wolfe *et al*., 2006 |
| *Penstemon rupicola* | Plantaginaceae | Grant, 1994 |
| *Penstemon subulatus* | Plantaginaceae | Grant, 1994 |
| *Penstemon superbus* | Plantaginaceae | Wolfe *et al*., 2006 |
| *Penstemon utahensis* | Plantaginaceae | Grant, 1994 |
| *Penstemon wrightii* | Plantaginaceae | Wolfe *et al*., 2006 |
| *Polemonium brandegeei* | Polemoniaceae | Grant, 1994 |
| *Polemonium pauciflorum* | Polemoniaceae | Grant, 1994 |
| *Ribes speciosum* | Grossulariaceae | Grant, 1994 |
| *Saltugilia splendens* San Bernhardino race | Polemoniaceae | Grant, 1994 |
| *Salvia coccinea* | Lamiaceae | Wester & Claßen-Bockhoff, 2007 |
| *Salvia greggii* | Lamiaceae | Wester & Claßen-Bockhoff, 2007 |
| *Salvia henryi* | Lamiaceae | Grant, 1994 |
| *Salvia lemmonii* | Lamiaceae | Grant, 1994 |
| *Salvia regla* | Lamiaceae | Wester & Claßen-Bockhoff, 2007 |
| *Salvia spathacea* | Lamiaceae | Grant, 1994 |
| *Sarcodes sanguinea* | Ericaceae | de Jesus Urtecho, 1987 |
| *Satureja mimuloides* | Lamiaceae | Grant, 1994 |
| *Scrophularia macrantha* | Scrophulariaceae | Grant, 1994 |
| *Silene californica* | Caryophyllaceae | Grant, 1994 |
| *Silene laciniata* | Caryophyllaceae | Grant, 1994 |
| *Silene regia* | Caryophyllaceae | Bertin, 1982 |
| *Silene rotundifolia* | Caryophyllaceae | Bertin, 1982 |
| *Silene serpenticola* | Caryophyllaceae | This study |
| *Silene virginica* | Caryophyllaceae | Fenster *et al.*, 2006 |
| *Spigelia marilandica* | Loganiaceae | Bertin, 1982 |
| *Stachys chamissonis* | Lamiaceae | Grant, 1994 |
| *Stachys ciliata* | Lamiaceae | Grant, 1994 |
| *Stachys coccinea* | Lamiaceae | Grant, 1994 |

**Table S6:** Hummingbird-adapted plant species of temperate South America. Clades or species for which divergence times have been inferred (as cited in Table 2) are marked in red.

| **Species** | **Family** | **Reference** |
| --- | --- | --- |
| *Asteranthera ovata* | Gesneriaceae | Aizen & Vázquez, 2006 |
| *Bomarea salsilla* | Alstroemeriaceae | Estades, 2003 |
| *Caiophora coronata* | Loasaceae | Arroyo *et al*., 1990 |
| *Campsidium valdivianum* | Bignoniaceae | Aizen & Vázquez, 2006 |
| *Colletia ulicina* | Rhamnaceae | Medan & Montaldo, 2005 |
| *Crinodendron hookerianum* | Elaeocarpaceae | Aizen & Vázquez, 2006 |
| *Cuminia eriantha* | Lamiaceae | Bernadello *et al*., 2001 |
| *Dendroseris litoralis* | Asteraceae | Schuchmann, 1999 |
| *Desfontainia spinosa* | Desfontainiaceae | Smith-Ramirez, 1993 |
| *Desmaria mutabilis* | Loranthaceae | Based on flower morphology and color |
| *Eccremocarpus scaber* | Bignoniaceae | Belmonte *et al.*, 1994 |
| *Embothrium coccineum* | Proteaceae | Aizen & Vázquez, 2006 |
| *Escallonia callcottiae* | Escalloniaceae | Bernadello *et al*., 2001 |
| *Escallonia rubra* | Escalloniaceae | Aizen & Vázquez, 2006 |
| *Fascicularia bicolor* | Bromeliaceae | Aizen & Vázquez, 2006 |
| *Fuchsia lycioides* | Onagraceae | Atsatt & Rundel, 1982 |
| *Fuchsia magellanica* | Onagraceae | Aizen & Vázquez, 2006 |
| *Greigia berteroi* | Bromeliaceae | Bernadello *et al*., 2001 |
| *Greigia landbeckii* | Bromeliaceae | Based on flower morphology and color |
| *Greigia pearcei* | Bromeliaceae | Based on flower morphology and color |
| *Greigia sphacelata* | Bromeliaceae | Based on flower morphology and color |
| *Hippeastrum uniflorum* | Amaryllidaceae | Arroyo *et al.*, 1990 |
| *Iochroma australe* | Solanaceae | Arroyo *et al*., 1987 |
| *Lapagaria rosea* | Philesiaceae | Aizen & Vázquez, 2006 |
| *Latua pubiflora* | Solanaceae | Mabberley, 2008 |
| *Lepechinia salviae* | Lamiaceae | Gutiérrez-Zamora, 2008 |
| *Lobelia bridgesii* | Campanulaceae | Based on flower morphology and color |
| *Lobelia excelsa* | Campanulaceae | Stacey, 2014 |
| *Lobelia polyphylla* | Campanulaceae | Based on flower morphology and color |
| *Lobelia tupa* | Campanulaceae | Estades, 2003 |
| *Mitraria coccinea* | Gesneriaceae | Aizen & Vázquez, 2006 |
| *Mutisia subulata* | Asteraceae | Based on flower morphology and color |
| *Nicotiana cordifolia* | Solanaceae | Bernadello *et al*., 2001 |
| *Notanthera heterophylla* | Loranthaceae | Aizen & Vázquez, 2006 |
| *Ochagavia carnea* | Bromeliaceae | Based on flower morphology and color |
| *Ochagavia elegans* | Bromeliaceae | Bernadello *et al*., 2001 |
| *Ochagavia litoralis* | Bromeliaceae | Based on flower morphology and color |
| *Ourisia coccinea* | Plantaginaceae | Based on flower morphology and color |
| *Ourisia polyantha* | Plantaginaceae | Based on flower morphology and color |
| *Ourisia ruellioides* | Plantaginaceae | Assumed by Arroyo & Penaloza, 1990 based on flower morphology and color |
| *Passiflora pinnatistipula* | Passifloraceae | Based on flower morphology and color |
| *Philesia magellanica* | Philesiaceae | Aizen & Vázquez, 2006 |
| *Puya coerulea* | Bromeliaceae | Jabaily & Sytsma, 2010 |
| *Puya venusta* | Bromeliaceae | Jabaily & Sytsma, 2010 |
| *Rhaphithamnus venustus* | Verbenaceae | Schuchmann, 1999 |
| *Sarmienta repens* | Gesneriaceae | Aizen & Vázquez, 2006 |
| *Schizanthus grahamii* | Solanaceae | Pérez *et al*., 2006 |
| *Sophora fernandeziana* | Fabaceae | Bernadello *et al*., 2001 |
| *Sophora masafuerana* | Fabaceae | Bernadello *et al*., 2001 |
| *Tristerix aphyllus* | Loranthaceae | Caballero *et al*., 2013 |
| *Tristerix corymbosus* | Loranthaceae | Smith-Ramirez, 1993 |
| *Tristerix tetrandus* | Loranthaceae | Fraga *et al*., 1997 |
| *Tristerix verticillatus* | Loranthaceae | Martínez-Harms *et al*., 2010 |
| *Tropaeolum speciosum* | Trophaeolaceae | Smith-Ramirez, 1993 |
| *Tropaeolum tricolor* | Trophaeolaceae | Smith-Ramirez, 1993 |
| *Vestia foetida* | Solanaceae | Knapp, 2010 |

**References for Tables S5 and S6**

**Abrol DP. 2012.** *Pollination Biology – Biodiversity Conservation and Agricultural Production.* Springer, Heidelberg.

**Aizen MA, Vázquez DP. 2006.** Flowering phenologies of hummingbird plants from the temperate forest of southern South America: is there evidence of competitive displacement? *Ecography* **29:** 357-366.

**Alford JD Anderson LC. 2002.** The taxonomy and morphology of *Macranthera flammea* (Orobanchaceae). *Sida, Contributions to Botany* **20:** 189-204.

**Amico GC, Vidal-Russell R, Nickrent DL. 2007.** Phylogenetic relationships and ecological speciation in the mistletoe *Tristerix* (Loranthaceae): The influence of pollinators, dispersers, and hosts. *American Journal of Botany* **94:** 558-567.

**Arriaga L, Rodriguez-Estrella R, Ortega-Rubio A. 1990.** Endemic hummingbirds and madrones of Baja: Are they mutually dependent? *Southwestern Naturalist* **35:** 76-79.

**Arroyo MTK, Penaloza A. 1990.** Genetic self-compatibility in a South American species of *Ourisia* (Scrophulariaceae). *New Zealand Journal of Botany* **28:** 467-470.

**Atsatt PR, Rundel PW. 1982.** Pollinator maintenance vs. fruit production: partitioned reproductive effort in subdioecious *Fuchsia lycioides*. *Annals of the Missouri Botanical Garden* **69:** 199-208.

**Bastida JM, Alcántara JM, Rey PJ, Vargas P, Herrera CM. 2010.** Extended phylogeny of *Aquilegia*: the biogeographical and ecological patterns of two simultaneous but contrasting radiations. *Plant Systematics and Evolution* **284:** 171-185.

**Bell CD, Soltis DE, Soltis PS. 2010.** The age and diversification of the angiosperms re-visited. *American Journal of Botany* **97:** 1296-1303.

**Belmonte Schwarzberg E. 1999.** Patrón de visita de *Bombus dahlbomii* (Apidae): Un abejorro que liba en *Eccremocarpus scaber* (Bignoniaceae) en Chile central. *Idesia* **17:** 73-77.

**Bernadello G, Aguilar R, Anderson GJ. 2004.** The reproductive biology of *Sophora fernandeziana* (Leguminosae), a vulnerable endemic species from Isla Robinson Crusoe. *American Journal of Botany* **91:** 198-206.

**Bernadello G, Anderson GJ, Stuessy TF, Crawford DJ. 2001.** A survey of floral traits, breeding systems, floral visitors, and pollination systems of the angiosperms of the Juan Fernandez Islands (Chile). *Botanical Review* **67:** 255-308.

**Berry PE, Hahn WJ, Sytsma KJ, Hall JC, Mast A. 2004.** Phylogenetic relationships and biogeography of *Fuchsia* (Onagraceae) based on noncoding nuclear and chloroplast DNA data. *American Journal of Botany* **91:** 601-614.

**Bertin RI. 1982.** Floral biology, hummingbird pollination and fruit production of trumpet creeper (*Campsis radicans*, Bignoniaceae). *American Journal of Botany* **69:** 122-134.

**Boyd AE. 2004.** Breeding system of *Macromeria viridiflora* (Boraginaceae) and geographic variation in pollinator assemblage. *American Journal of Botany* **91:** 1809-1813.

**Carpenter FL. 1978.** A spectrum of nectar-eater communities. *American Zoologist* **18:** 809-819.

**Caruso CM, Peterson SB, Ridley CE. 2003.** "Natural selection on floral traits of *Lobelia* (Lobeliaceae): spatial and temporal variation". *American Journal of Botany* **90:** 1333–40.

**Chacón J, Renner SS. 2014**. Assessing model sensitivity in ancestral area reconstruction using Lagrange: A case study using the Colchicaceae family. *Journal of Biogeography* **41:** 1414-1427.

**Cohen JI. 2012.** Continuous characters in phylogenetic analyses: Patterns of corolla tube length evolution in *Lithospermum* L. (Boraginaceae). *Biological Journal of the Linnean Society* **107:** 442-457.

**Côrtes ALA. 2013.** Sistemática e biogeografia da linhagem *Tetramerium* (Acanthaceae) na América do Sul. PhD-thesis, Universidade estadual de Feira de Santana, Brazil.

**Daniel TF. 1982.** *Anisacanthus andersonii* (Acanthaceae), a new species from northwestern Mexico. *Bulletin of the Torrey Botanical Club* **109:** 148-151.

**De Jesus Urtecho R. 1987.** The floral biology, hummingbird pollination, and potential breeding systems of the snow plant, *Sarcodes sanguinea* Torr. (Ericaceae). Master thesis, St. Bonaventure University.

**Dennis JV, Tekulsky M. 1991.** *How to attract butterflies and hummingbirds?* Ortho Books, San Ramon.

**Drew BT, Sytsma KJ. 2012.** Phylogenetics, biogeography, and staminal evolution in the tribe Mentheae (Lamiaceae). *American Journal of Botany* **99:** 933-953.

**Drew BT, Sytsma KJ. 2013.** The South American radiation of *Lepechinia* (Lamiaceae): phylogenetics, divergence times and evolution of dioecy. *Botanical Journal of the Linnean Society* **171:** 171-190.

**Drummond AJ, Rambaut A. 2007.** BEAST: Bayesian evolutionary analysis by sampling trees. *BMC Evolutionary Biology* **7:** 214.

**Duffield WJ. 1971.** Pollination ecology of *Castilleja* in Mount Rainer National Park. *Ohio Journal of Science* **72:** 110-114.

**Estes JR, Hall PM. 1975.** Pollination of *Ipomopsis rubra* (Polemoniaceae) by Ruby-throated hummingbirds. Bulletin of the Torrey Botanical Club **102:** 413-415.

**Fenster MC, Cheely G, Dudash MR, Reynolds RJ. 2006.** Nectar reward and advertisement in hummingbird-pollinated *Silene virginica* (Caryophyllaceae). *American Journal of Botany* **93:** 1800-1907.

**Filipowicz N, Renner SS. 2012.** *Brunfelsia* (Solanaceae): A genus evenly divided between South America and radiations on Cuba and other Antillean islands. *Molecular Phylogenetics and Evolution* **64:** 1-11.

**Givnish TJ, Barfuss MHJ, Van Ee B, Riina R, Schulte K, Horres R, Gonsiska PA, Jabaily RS, Crayn DM, Smith JAC, Winter K, Brown GK, Evans TM, Holstm BK, Luther H, Till W, Zizka G, Berry PE, Sytsma KJ. 2014.** Adaptive radiation, correlated and contingent evolution, and net species diversification in Bromeliaceae. *Molecular Phylogenetics and Evolution* **71:** 55-78.

**Grant V. 1994.** Historical development of ornithophily in the western North American flora. *Proceedings of the Natural Academy of Science U.S.A.* **91:** 10407-10411.

**Gussarova G, Popp M, Vitek E, Brochmann C. 2008.** Molecular phylogeny and biogeography of the bipolar *Euphrasia* (Orobanchaceae): Recent radiations in an old genus. *Molecular* *Phylogenenetics and Evolution* **48:** 444-460.

**Hagen SH. 1941.** A revision of the North American species of the genus *Anisacanthus*. *Annals of the Missouri Botanical Garden* **28:** 385-408.

**Hardy NB, Cook LG. 2012.** Testing for ecological limitation of diversification: A case study using parasitic plants. *American Naturalist* **180:** 438-449.

**Holmquist JP-H, Manktelow M, Daniel TF. 2005.** Wing pollination by bees in *Mexacanthus* (Acanthaceae)? *Acta Botánica Mexicana* **71:** 11-17.

**Hornung-Leoni CT, González-Gómez PL, Troncoso AJ. 2013.** Morphology, nectar characteristics and avian pollinators in five Andean *Puya* species (Bromeliaceae). *Acta Oecologica* **51:** 54-61.

**Jabaily RS, Sytsma KJ. 2010.** Phylogenetics of *Puya* (Bromeliaceae): Placement, major lineages, and evolution of Chilean species. *American Journal of Botany* **97:** 337-356.

**Jabbour F, Renner SS. 2012.** A phylogeny of Delphinieae (Ranunculaceae) shows that *Aconitum* is nested within *Delphinium* and that late Miocene transitions to long life cycles in the Himalayas and southwest China coincide with bursts in diversification. *Molecular Phylogenetics and Evolution* **62:** 928-942.

**James DW. 1972.** Pollination ecology of *Castilleja* in Mount Rainer National Park. *Ohio Journal of Science* **72:** 110-114.

**Kay KM, Whittall JB, Hodges SA. 2006.** A survey of nuclear ribosomal internal transcribed spacer substitution rates across angiosperms: an approximate molecular clock with life history effects. *BMC Evolutionary Biology* **6:** 36, doi:10.1186/1471-2148-6-36.

**Kim S-C, Chunghee L, Mejías JA. 2007.** Phylogenetic analysis of chloroplast DNA *matK* gene and ITS of nrDNA sequences reveals polyphyly of the genus *Sonchus* and new relationships among the subtribe Sonchinae (Asteraceae: Cichorieae). *Molecular Phylogenetics and Evolution* **44:** 578-597.

**Lara-Rodríguez NZ, Díaz-Valenzuela R, Martínez-García V, Mauricio-Lopéz E, Anaid-Diaz S, Valle OI, Fisher-de Léon AD, Lara C, Ortiz-Pulido R. 2012.** Redes de interaccíon colibrí-planta del central-ests México. *Revista Mexicana de Biodiversidad* **83:** 569-577.

**Lott TA, Manchester SR, Dilcher DL. 1998.** A unique and complete polemoniaceous plant from the middle Eocene of Utah, USA. *Review of Palaeobotany and Palynology* **104:** 39-49.

**Martínez-Harms J, Palacios AG, Márquez N, Estay P, Arroyo MTK. Mpodozis J. 2010.** Can red flowers be conspicuous to bees? *Bombus dahlbomii* and South American temperate forest flowers as a case in point. *Journal of Experimental Biology* **213:** 564-571.

**Marx HE, O´Leary N, Yuan Y-W, Lu-Irving P, Tank DC, Múlgura ME, Olmstead RG. 2010.** A molecular phylogeny and classification of the Verbenaceae. *American Journal of Botany* **97:** 1647-1663.

**McDonald C:** Forest service: [http://www.fs.fed.us/wildflowers/plant-of-the-week/agastache_rupestris.shtml](http://www.fs.fed.us/wildflowers/plant-of-the-%09week/agastache_rupestris.shtml) January 2013.

**McGuire JA, Witt CC, Remsen JV, Corl A, Rabowsky D, Altshuler DL, Dudley R. 2014.** Molecular phylogenetics and diversification of hummingbirds. *Current Biology* **24:** doi:10.1016/j.cub.2014.03.016.

**Medan D, Montaldo NH. 2005.** Ornithophily in the Rhamnaceae: The pollination of the Chilean endemic *Colletia ulicina*. *Flora* **200:** 339-344.

**Montero-Castro JC, Delgado-Salinas A, de Luna E, Eguiarte LE. 2006.** Phylogenetic analysis of *Cestrum* section *Habrothamnus* (Solanaceae) based on plastid and nuclear DNA sequences. *Systematic Botany* **31:** 843-850.

**Olmstead RG, Bohs L, Migid HA, Santiago-Valentin E, Garcia VF, Collier SM. 2008.** A molecular phylogeny of the Solanaceae. *Taxon* **57:** 1159-1181.

**Olmstead RG, Zjhra ML, Lohmann LG, Grose SO, Eckert AJ. 2009.** A molecular phylogeny and classification of Bignoniaceae. *American Journal of Botany* **96:** 1731-1743.

**Pellmyr O, Augenstein EJ.** **1997.** Pollination biology of *Hersperaloe parviflora*. *Southwestern*  *Naturalist* **42:** 182-187.

**Pérez F, Arroyo MTK, Medel R, Hershkovitz MA. 2006.** Ancestral reconstruction of flower morphology and pollination systems in *Schizanthus* (Solanaceae). *American Journal of* *Botany* **93:** 1029-1038.

**Porter JM, Johnson LA, Wilken D. 2010.** Phylogenetic systematics of *Ipomopsis* (Polemoniaceae): Relationships and divergence times estimated from chloroplast and nuclear DNA sequences. *Systematic Botany* **35:** 181-200.

**Rambaut A, Drummond AJ. 2007.** Tracer v1.4. <http://beast.bio.ed.ac.uk/Tracer>.

**Reid S, Cornelius C, Barbosa O, Meynard C, Silva-García C, Marquet PA. 2002.** Conservation of temperate forest birds in Chile: implications from the study of an isolated forest relict. *Biodiversity and Conservation* **11:** 1975-1990.

**Richardson JE, Pennington RT, Pennington TD, Hollingsworth PM. 2001.** Rapid diversification of a species rich genus of neotropical rain forest trees. *Science* **293:** 5538.

**Roy MS, Torres-Mura JC, Hertel F. 1998.** Evolution and history of hummingbirds (Aves: Trochilidae) from the Juan Fernandez Islands, Chile. *Ibis* **140:** 265-273.

**Ruiz E, Crawford DJ, Stuessy TF, Gonzalez F, Samuel R, Becarra J, Silva M. 2004.** Phylogenetic relationships and genetic divergence among endemic species of *Berberis, Gunnera, Myrceugenia* and *Sophora* of the Juan Fernandez Islands (Chile) and their continental progenitors based on isozymes and nrITS sequences. *Taxon* **53:** 321-332.

**Särkinen TE, Newman MF, Maas PJM, Maas H, Poulsen AD, Harris DJ, Richardson RD, Clark A, Hollingsworth M, Pennington RT. 2007.** Recent oceanic long-distance dispersal and divergence in the amphi-Atlantic rain forest genus *Renealmia* L.f. (Zingiberaceae). *Molecular Phylogenenetics and Evolution* **44:** 968-980.

**Scheunert A, Heubl G. 2011.** Phylogenetic relationships among New World *Scrophularia* L. (Scrophulariaceae): new insights inferred from DNA sequence data. *Plant Systematics and Evolution* **291:** 69-89.

**Schnabel A, Wendel JF. 1998.** Cladistic biogeography of *Gleditsia* (Leguminosae) based on *ndhF* and *rpl16* chloroplast gene sequences. *American Journal of Botany* **85:** 1753-1765.

**Schuchmann K-L 1999.** *Family Trochilinae (Hummingbirds)*. In: Del Hoyo J, Elliot A Sargatal J (eds) Handbook of Birds of the World. Vol. 5: Barn-owls to hummingbirds. Barcelona, pp 468-680.

**Schultheis LM, Donoghue MJ. 2004.** Molecular phylogeny and biogeography of *Ribes* (Grossulariaceae), with an emphasis on gooseberries (subg. *Grossularia*). *Systematic Botany* **29:** 77-96.

**Smith SA, Donoghue MJ. 2010.** Combining historical biogeography with niche modelling in the *Caprifolium* clade of *Lonicera* (Caprifoliaceae, Dipsacales). *Systematic Botany* **59:** 322-341.

**Smith-Ramirez C. 1993.** Los picaflores y su recurso floral en el bosque templado de la isla de Chiloé, Chile. *Revista Chilena de Historia Natural* **66:** 65-73.

**Soria-Hernanz DF, Fiz-Palacios O, Braverman JM, Hamilton MB. 2008.** Reconsidering the generation time hypothesis based on nuclear ribosomal *ITS* sequence comparisons in annual and perennial angiosperms. *BMC Evolutionary Biology* **8:** doi:10.1186/1471-2148-8-344.

**Steiner KE. 1979.** Passerine pollination of *Erythrina megistophylla* Diels (Fabaceae). *Annals of the* *Missouri Botanical Garden* **66:** 490-502.

**Stiles FG. 1973.** *Food supply and the annual cycle of the Anna hummingbird*. University of California Press, Berkeley.

**Tank** **DC, Olmstead RG. 2008.** From annuals to perennials: phylogeny of subtribe Castillejinae (Orobanchaceae). *American Journal of Botany* **95:** 608-635.

**Ulaszek EF. 2001.** Todsen’s Pennyroyal *(Hedeoma todsenii) -* Revised recovery plan. *U.S. Fish and Wildlife Service New Mexico Ecological Services Field Office,* Albuquerque, New Mexico.

**Valdivia CE, Simonetti JA, Henríquez CA. 2006.** Depressed pollination of *Lapageria rosea* Ruiz et Pav. (Philesiaceae) in the fragmented temperate rainforest of southern South America. *Biodiversity and Conservation* **15:** 1845-1856.

**van Devender TR, Calder WA, Krebbs K, Reina AL, Russell SM, Russell RO. 2004.** *Hummingbird plants and potential nectar corridors of the Rufous Hummingbird in Sonora, Mexico*. In: Nabhan GP. Conserving migratory pollinators and nectar corridors in western North America. University of Arizona Press, Phoenix.

**Vargas P, Carrió E, Guzmán B, Amat E, Güemes J. 2009.** A geographical pattern of *Antirrhinum* (Scrophulariaceae) speciation since the Pliocene based on plastid and nuclear DNA polymorphisms. *Journal of Biogeography* **36:** 1297-1312.

**Vidal-Russell R, Nickrent DL. 2008.** The first mistletoes: Origin of aerial parasitism in Santalales. *Molecular Phylogenetics and Evolution* **47:** 523-537.

**Wester P, Claßen-Bockhoff R. 2007.** Floral diversity and pollen transfer mechanisms in bird-pollinated *Salvia* species. *Annals of Botany* **100:** 401-421.

**Whitten WM. 1981.** Pollination ecology of *Monarda didyma*, *M. clinopodia*, and hybrids (Lamiaceae) in the Southern Appalachian Mountains. *American Journal of Botany* **68:** 435-442.

**Williamson SL. 2001.** Hummingbirds of North America. Houghton Mifflin company, Boston, New York.

**Wolfe AD, Randale CP, Datwyler SI, Morawetz JJ, Arguedas N, Diaz J. 2006.** Phylogeny, taxonomic affinities, and biogeography of *Penstemon* (Plantaginaceae) based on ITS and cpDNA sequence data. *American Journal of Botany* **93:** 1699-1713.

**Woo VL, Funke MM, Smith FJ, Lockhart PJ, Garlock-Jones PJ. 2011.** New World origin of southwest Pacific Gesneriaceae: Multiple movements across and within the South Pacific. *International Journal of Plant Sciences* **172:** 434-457.

**Wood TE, Nakazato T. 2009.** Investigating species boundaries in the Giliopsis group of *Ipomopsis* (Polemoniaceae): Strong discordance among molecular and morphological markers. *American Journal of Botany* **96:** 853-861.

**Xiang Q-Y, Soltis DE, Soltis PS, Manchester SR, Crawford DJ. 2000.** Timing the eastern Asian–eastern North American floristic disjunction: Molecular clock corroborates paleontological estimates. *Molecular Phylogenetics and Evolution* **15:** 462-472.

**Table S7**. Sister taxa in which one member of a pair is pollinated by hummingbirds, the other is not, together with their species numbers.

| Hummingbird-pollinated clade | Species number | Time of sister pair divergence [my ago] | Not hummingbird-pollinated clade | Species number |
| --- | --- | --- | --- | --- |
| *Monarda didyma* | 1 | 0.56 | *Monarda stipitatoglandulosa* | 1 |
| *Arbutus peninsularis* | 1 | 0.81 | *Arbutus xalapensis* | 1 |
| *Lithospermum leonotis* | 1 | 1 | *Lithospermum nelsonii* | 1 |
| *Keckiella cordifolia* | 1 | 1.19 | *Keckiella antirrhinoides* | 1 |
| *Keckiella ternata* clade | 3 | 1.39 | *Keckiella breviflora* | 1 |
| *Ribes speciorum* | 1 | 1.45 | *Ribes californicum* | 1 |
| *Ipomopsis tenuifolia* | 1 | 1.45 | *Ipomopsis guttata/effusa* | 2 |
| *Lithospermum macromeria* | 1 | 1.6 | *Lithospermum multiflorum* | 1 |
| *Anisacanthus sensu stricto* | 5 | 1.92 | *Tetrameria* clade | 28 |
| *Collomia rawsoniana* | 1 | 2.12 | *Collomia linearis* | 1 |
| *Aquilegia* clade | 10 | 2.52 | *Aquilegia coeruleum* | 1 |
| *Lithospermum johnstonii* | 1 | 2.55 | *Lithospermum exsertum* | 1 |
| *Delphinium cardinale* | 1 | 2.93 | *Delphinium decorum* clade | 3 |
| *Campsis radicans* | 1 | 3.26 | *Campsis grandiflora* | 1 |
| *Scrophularia macrantha* | 1 | 3.57 | *Scrophularia laevis* | 1 |
| *Ipomopsis aggregata* clade | 22 | 4.73 | *Ipomopsis polycladon* | 1 |
| *Castilleja miniata* clade | >23 | 5.14 | *Castilleja tenuis* clade | 4 |
| *Lithospermum notatum/flavum* | 2 | 8.25 | *Lithospermum obovatum* clade | 30 |
| *Lonicera* North American clade | 6 | 9.19 | *Lonicera hispidula* clade | 3 |
| *Tourretia/Eccremocarpus* | 3 | 26.82 | Rest Bignoniaceae | >50 |
| *Desfontainia spinosa* | 1 | 39.53 | *Columella* | 2 |
| *Puya venusta* | 1 | 0.68 | *Puya alpestris* | 1 |
| *Puya coerulea* | 1 | 1.68 | *Puya gilmartinae* | 1 |
| *Rhaphithamnus venustus* | 1 | 1.96 | *Rhaphithamnus spinosus* | 1 |
| *Schizanthus grahamii* | 1 | 1.98 | *Schizanthus hookeri* | 1 |
| *Dendroseris litoralis* | 1 | 3.39 | *Dendroseris berteroana/pinnata* | 2 |
| *Lepechinia salviae* | 1 | 3.65 | *Lepechinia chamaedryoides* | 1 |
| *Cuminia eriantha* | 1 | 4.07 | *Kurzamra pulchella* | 1 |
| *Campsidium valdivianum* | 1 | 12.8 | *Pandorea jasminoides* | 1 |
| *Vestia foetida* | 1 | 12.85 | *Sessea stipulata* | 1 |
| *Latua pubiflora* | 1 | 14.11 | *Atropa* clade | >30 |
| Chilean Gesneriaceae | 4 | 26.2 | *Negria* clade | 5 |
| Philesiaceae | 2 | 58.8 | *Ripogonaceae* | 6 |

**Figure S1a.** Chronogram for 221 species of hummingbirds, rooted on 5 species of swifts, based on 4022 nucleotides of nuclear and mitochondrial DNA (*Materials and Methods*) analyzed under a strict clock model calibrated with a 47.5 my-old hummingbird-like fossil (red star). Numbers above branches are node ages (my). The North American species are marked in blue, the southern South America species in red, and the blue circle marks the crown group of the North American clade. The temperate South American *Oreotrochilus leucopleurus* is missing, and for this species we used the stem age of *Oreotrochilus* (blue circle). The photo (by Steve Garvie, www.wikipedia.org) shows *Lophornis ornatus* at *Stachytarpheta* spec. (Verbenaceae) flowers. The map shows the biogeographic regions used in the ancestral area reconstructions. Note that the biogeographic analysis was done including all species.

**Figure S1b.** Chronogram from the same matrix as used for Fig. S1a analyzed under a UCLN relaxed clock model calibrated with a mitochondrial substitution rate (*Materials and Methods*). Numbers above branches are node ages (my) and bars at nodes with ≥98% posterior probability indicate the 95% confidence intervals on the estimated times. The coloring of bird species is as in Fig. S1a.

**Figure S2a.** Chronogram for *Aquilegia* (Ranunculaceae) obtained by Bastida *et al.* (2010) under a Bayesian relaxed clock model applied to 34 accessions and 2486 aligned nucleotides of plastid and nuclear DNA sequences, using a fossil calibration. Bars at nodes with ≥98% posterior probability indicate the 95% confidence intervals on the estimated times. Numbers above branches are node ages (my), the red circle indicates the cross validation point, red bars hummingbird-pollinated species, blue bars moth-pollinated species, green bars fly-pollinated species, white bars hymenoptera-pollinated species, red stars the stem ages and the yellow star the crown age of the hummingbird-pollinated clade, indicated by red branches.

**Figure S2b.** Chronogram for *Castilleja* (Orobanchaceae) obtained under a Bayesian strict clock model applied to 81 accessions and 3257 aligned nucleotides of nuclear DNA sequences. Bars at nodes with ≥98% posterior probability indicate the 95% confidence intervals on the estimated times. Numbers above branches are node ages (my), the red circle indicates the cross validation point, red bars hummingbird-pollinated species, white bars insect-pollinated species, the red stars the stem ages and the yellow stars the crown ages of the hummingbird-pollinated clades, indicated by red branches.

**Figure S2c.** Chronogram for *Ipomopsis* and *Collomia* (Polemoniaceae) obtained under a Bayesian relaxed clock model applied to 100 accessions and 1234 aligned nucleotides of plastid and nuclear DNA sequences. Bars at nodes with ≥98% posterior probability indicate the 95% confidence intervals on the estimated times. Numbers above branches are node ages (my), the red circle indicates the cross validation point, red bars hummingbird-pollinated species, blue bars moth-pollinated species, green bars butterfly-pollinated species, white bars hymenoptera-pollinated species, red stars the stem ages and the yellow star the crown age of the hummingbird-pollinated clade/species, indicated by red branches.

**Figure S2d.** Chronogram for *Keckiella* (Plantaginaceae) obtained under a Bayesian strict clock model applied to 17 accessions and 1359 aligned nucleotides of nuclear DNA sequences. Bars at nodes with ≥98% posterior probability indicate the 95% confidence intervals on the estimated times. Numbers above branches are node ages (my), the red circle indicates the cross validation point, red bars hummingbird-pollinated species, white bars insect-pollinated species, red stars the stem ages and a yellow star the crown age of the hummingbird-pollinated clade/species, indicated by red branches.

**Figure S2e.** Chronogram for *Lithospermum* (Boraginaceae) obtained by Cohen (2012) under a Bayesian relaxed clock model applied to 42 accessions and about 10118 aligned nucleotides of plastid and nuclear DNA sequences using a fossil calibration. Bars at nodes with ≥98% posterior probability indicate the 95% confidence intervals on the estimated times. Numbers above branches are node ages (my), the red circle indicates the cross validation point, red bars hummingbird-pollinated species, blue bars moth-pollinated species, white bars hymenoptera-pollinated species, the red stars the stem ages and the yellow star the crown age of the hummingbird-pollinated clade/species, indicated by red branches.

**Figure S2f.** Chronogram for *Lonicera* (Caprifoliaceae) obtained by Smith and Donoghue (2010) under a Bayesian clock model applied to 17 accessions and about 1800 aligned nucleotides of plastid and nuclear DNA sequences, using a secondary calibration. Bars at nodes with ≥98% posterior probability indicate the 95% confidence intervals on the estimated times. Numbers above branches are node ages (my), the red circle indicates the cross validation point, red bars hummingbird-pollinated species, blue bars moth-pollinated species, white bars hymenoptera-pollinated species, the red stars the stem ages and the yellow star the crown age of the hummingbird-pollinated clade/species, indicated by red branches.

**Figure S2g.** Chronogram for *Monarda* (Lamiaceae) obtained under a Bayesian relaxed clock model applied to 19 accessions and 970 aligned nucleotides of plastid and nuclear DNA sequences. Bars at nodes with ≥98% posterior probability indicate the 95% confidence intervals on the estimated times. Numbers above branches are node ages (my), the red circle indicates the cross validation point, red bar hummingbird-pollinated species, white bars hymenoptera-pollinated species, and the red star the stem age of the hummingbird-pollinated species, indicated by a red branch.

**Figure S2h**. Chronogram for *Ribes* (Grossulariaceae) obtained under a Bayesian relaxed clock model applied to 52 accessions and 1259 aligned nucleotides of plastid and nuclear DNA sequences. Bars at nodes with ≥98% posterior probability indicate the 95% confidence intervals on the estimated times. Numbers above branches are node ages (my), the red circle indicates the cross validation point, red bar hummingbird-pollinated species, white bars insect-pollinated species, and the red star the stem age of the hummingbird-pollinated species, indicated by a red branch.

**Figure S3a:** Chronogram for the *Campsidium* (Bignoniaceae) obtained under a Bayesian relaxed clock model applied to 88 accessions and 3327 aligned nucleotides of chloroplast DNA sequences. Bars at nodes with ≥98% posterior probability indicate the 95% confidence intervals on the estimated times. Numbers above branches are node ages (my), the red circle indicates the cross validation point, red bars hummingbird-pollinated species, brown bars sunbird-pollinated species, green bars butterfly-pollinated species, missing bars bee-pollinated species, the red stars the stem ages and the yellow star the crown age of the hummingbird-pollinated clade(s), indicated by red branches. Pollinators were only inferred for the taxa within the box.

**Figure S3b:** Chronogram for *Dendroseris* (Asteraceae) obtained under a Bayesian relaxed clock model applied to 65 accessions and 580 aligned nucleotides of nuclear DNA sequences. Bars at nodes with ≥98% posterior probability indicate the 95% confidence intervals on the estimated times. Numbers above branches are node ages (my), the red circle indicates the cross validation point, red bar hummingbird-pollinated species, white bars bee-pollinated species, and the red star the stem age of the hummingbird-pollinated species, indicated by a red branch.

**Figure S3c:** Chronogram for *Latua* (Solanaceae) obtained under a Bayesian relaxed clock model applied to 183 accessions and 3040 aligned nucleotides of plastid and nuclear DNA sequences. Bars at nodes with ≥98% posterior probability indicate the 95% confidence intervals on the estimated times. Numbers above branches are node ages (my), the red circle indicates the cross validation point, red bars hummingbird-pollinated species, white bars insect-pollinated species, the red stars the stem ages and the yellow star the crown age of the hummingbird-pollinated clades, indicated by a red branch. For the ancestral pollinator reconstruction (within the box) we only differentiate between hummingbird and insect pollination due to lack of detailed information on pollinators in many species.

**Figure S3d:** Chronogram for *Puya* (Bromeliaceae) obtained under a Bayesian strict clock model applied to 64 accessions and 2553 aligned nucleotides of chloroplast and nuclear DNA sequences. Bars at nodes with ≥98% posterior probability indicate the 95% confidence intervals on the estimated times. Numbers above branches are node ages (my), the red circle indicates the cross validation point, red bars hummingbird-pollinated species, light pink bars perching bird-pollination, and red stars the stem ages of the hummingbird-pollinated species, indicated by red branches.

**Figure S3e:** Chronogram for *Rhaphithamnus* (Verbenaceae) obtained under a Bayesian relaxed clock model applied to 46 accessions and 3065 aligned nucleotides of plastid DNA sequences. Bars at nodes with ≥98% posterior probability indicate the 95% confidence intervals on the estimated times. Numbers above branches are node ages (my), the red circle indicates the cross validation point, the red bar hummingbird-pollinated species (all other species are insect pollinated), and the red star the stem age of the hummingbird-pollinated species, indicated by red branches.

**Figure S3f:** Chronogram for *Schizanthus* (Solanaceae) obtained under a Bayesian relaxed clock model applied to 13 accessions and 1566 aligned nucleotides of plastid DNA sequences. Bars at nodes with ≥98% posterior probability indicate the 95% confidence intervals on the estimated times. Numbers above branches are node ages (my), the red circle indicates the cross validation point, red bar hummingbird-pollinated species, blue bars moth-pollinated species, white bars bee-pollinated species, and the red star the stem age of the hummingbird-pollinated species, indicated by red branches.

**Figure S3g:** Chronogram for *Tristerix* (Loranthaceae) obtained under a Bayesian relaxed clock model applied to 12 accessions and 2176 aligned nucleotides of plastid and nuclear DNA sequences. Bars at nodes with ≥98% posterior probability indicate the 95% confidence intervals on the estimated times. Numbers above branches are node ages (my), the red circle indicates the cross validation point, red bars hummingbird-pollinated species, white bars bee-pollinated species, the red star the stem age and the yellow star the crown age of the hummingbird-pollinated clade, indicated by red branches.

**Figure S3h:** Chronogram for *Vestia* (Solanaceae) obtained under a Bayesian relaxed clock model applied to 37 accessions and 3492 aligned nucleotides of plastid DNA sequences. Bars at nodes with ≥98% posterior probability indicate the 95% confidence intervals on the estimated times. Numbers above branches are node ages (my), the red circle indicates the cross validation point, red bars hummingbird-pollinated species, blue bars moth-pollinated species, missing bars bee-pollinated species, red stars the stem ages and yellows star the crown ages of the hummingbird-pollinated clades, indicated by a red branch.
